# Experience Sampling and Programmed Intervention Method and System for Planning, Authoring, and Deploying Mobile Health Interventions: Design and Case Reports

**DOI:** 10.2196/24278

**Published:** 2021-07-12

**Authors:** Bruna Carolina Rodrigues Cunha, Kamila Rios Da Hora Rodrigues, Isabela Zaine, Elias Adriano Nogueira da Silva, Caio César Viel, Maria Da Graça Campos Pimentel

**Affiliations:** 1 Federal Institute of Education, Science and Technology of São Paulo Capivari Brazil; 2 Institute of Mathematics and Computer Sciences University of São Paulo São Carlos Brazil; 3 Sidia Institute of Science and Technology Manaus Brazil

**Keywords:** mobile apps, mHealth, intervention, experience sampling, method, monitoring, Experience Sampling and Programmed Intervention Method, experience sampling method, ecological momentary assessment, just-in-time adaptive intervention

## Abstract

**Background:**

Health professionals initiating mobile health (mHealth) interventions may choose to adapt apps designed for other activities (eg, peer-to-peer communication) or to employ purpose-built apps specialized in the required intervention, or to exploit apps based on methods such as the experience sampling method (ESM). An alternative approach for professionals would be to create their own apps. While ESM-based methods offer important guidance, current systems do not expose their design at a level that promotes replicating, specializing, or extending their contributions. Thus, a twofold solution is required: a method that directs specialists in planning intervention programs themselves, and a model that guides specialists in adopting existing solutions and advises software developers on building new ones.

**Objective:**

The main objectives of this study are to design the Experience Sampling and Programmed Intervention Method (ESPIM), formulated toward supporting specialists in deploying mHealth interventions, and the ESPIM model, which guides health specialists in adopting existing solutions and advises software developers on how to build new ones. Another goal is to conceive and implement a software platform allowing specialists to be users who actually plan, create, and deploy interventions (ESPIM system).

**Methods:**

We conducted the design and evaluation of the ESPIM method and model alongside a software system comprising integrated web and mobile apps. A participatory design approach with stakeholders included early software prototype, predesign interviews with 12 health specialists, iterative design sustained by the software as an instance of the method’s conceptual model, support to 8 real case studies, and postdesign interviews.

**Results:**

The ESPIM comprises (1) a list of requirements for mHealth experience sampling and intervention-based methods and systems, (2) a 4-dimension planning framework, (3) a 7-step-based process, and (4) an ontology-based conceptual model. The ESPIM system encompasses web and mobile apps. Eight long-term case studies, involving professionals in psychology, gerontology, computer science, speech therapy, and occupational therapy, show that the method allowed specialists to be actual users who plan, create, and deploy interventions via the associated system. Specialists’ target users were parents of children diagnosed with autism spectrum disorder, older persons, graduate and undergraduate students, children (age 8-12), and caregivers of older persons. The specialists reported being able to create and conduct their own studies without modifying their original design. A qualitative evaluation of the ontology-based conceptual model showed its compliance to the functional requirements elicited.

**Conclusions:**

The ESPIM method succeeds in supporting specialists in planning, authoring, and deploying mobile-based intervention programs when employed via a software system designed and implemented according to its conceptual model. The ESPIM ontology–based conceptual model exposes the design of systems involving active or passive sampling interventions. Such exposure supports the evaluation, implementation, adaptation, or extension of new or existing systems.

## Introduction

Many factors impact the adoption of mobile health (mHealth) tools by professionals and their target population, as observed in Australia [1], Canada [2], USA [3,4], and in Europe [5,6]. As an example, clinicians’ concerns when considering an mHealth tool include usefulness, ease of use, compatibility, technical issues, content, personalization, convenience, strict data privacy, workload, workflow, communication, management support, and policies [7-9]. Such themes align with those highlighted by Chinese public hospitals’ managers [10], including perceived ease of use, system security and reliability, top management support, and government policy.

Toward employing mHealth interventions, professionals can generally choose among 3 options: using apps designed for other activities such as peer-to-peer communication, using purpose-built apps specialized in the required intervention, or using apps based on methods such as the experience sampling method (ESM) [11-13], and its descendent ecological momentary assessment (EMA) [14], including those exploring just-in-time adaptive interventions (JITAIs) [15]. The first alternative allows professionals to adapt their protocols to take advantage of popular apps [16] and to employ conventional SMS text messaging usually available to the underprivileged [17-19]. However, because interventions may require sending or collecting multiple types of questions and media and demand careful planning [20], deploying nonspecialized apps demands both adaptations in the protocol and overcoming obstacles when monitoring progress.

The second alternative led to the design of a wide range of mHealth-specialized apps [21-24] that enable reproducing interventions accurately. Their design engenders a dependency relationship between specialists and software developers. Moreover, specialized apps have little potential for reuse.

The third alternative involves using apps based on methods such as the ESM and EMA, as in the works surveyed by van Berkel et al [25]. Examples include studies [26-30] that employed the LifeData [31], the movisensXS [32], or the Mobile EMA [33] systems based on data collection methods. Additionally, ecological momentary interventions (EMIs) or JITAIs support interventions involving contextual data used for personalization according to users’ needs [15,34,35].

In a complementary approach, if professionals were able to create their own apps [36], they could focus on the methodological processes of their work. While the ESM methods offer important guidance, current systems [31-33] do not expose their design at a level that promotes replicating, specializing, or extending their contributions as demanded in many areas. Thus, a twofold solution is required: a method that directs specialists in planning an mHealth intervention program themselves, and a model that guides specialists in adopting existing solutions while advising software developers on building new ones.

The 2 main objectives of this study are to design the Experience Sampling and Programmed Intervention Method (ESPIM), formulated toward supporting specialists in deploying mHealth interventions, and the ESPIM model, which guides specialists in adopting existing solutions and advises software developers on how to build new ones. A subsidiary goal is to conceive and implement a software platform allowing specialists to be users who plan, create, and deploy interventions (ESPIM system).

## Methods

### Overview

**Figure 1:**
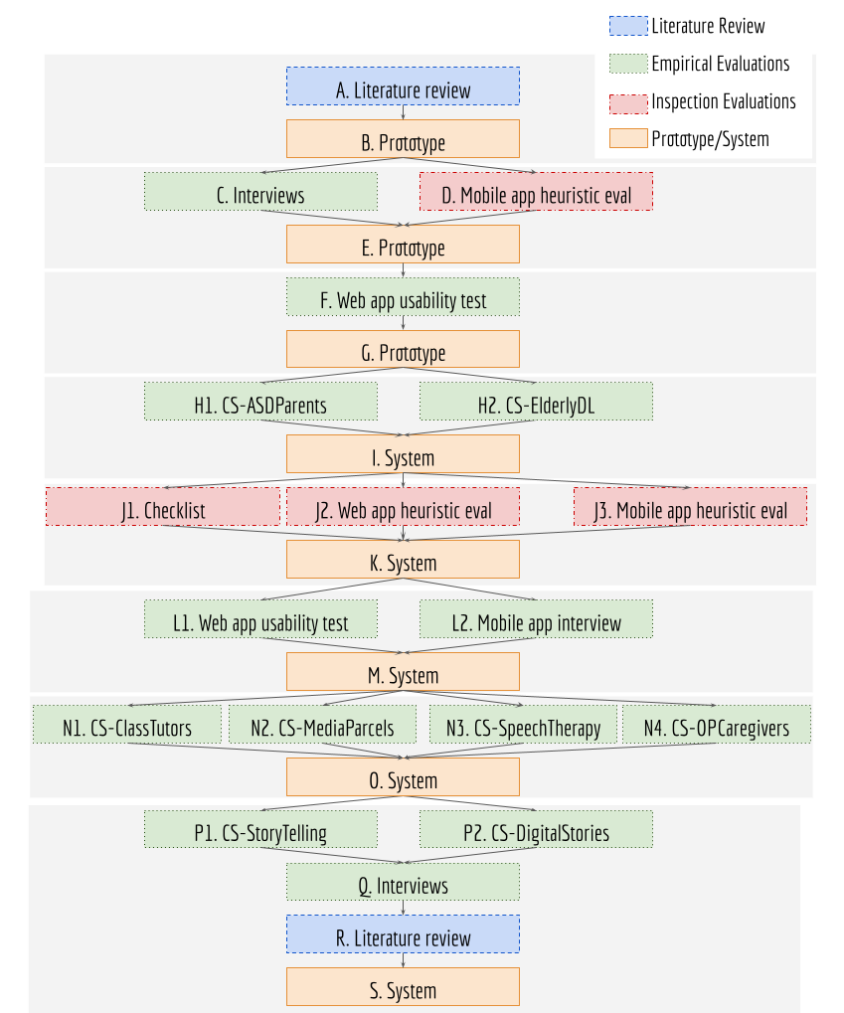
Study Methods and Workflow. CS: case study.

For designing the mobile-based ESPIM method, we adopted an iterative approach of co-design considering the participatory design practices [37,38]. Besides continuous review of state-of-the-art literature, the procedures adopted encompass early software prototype and predesign interviews with health specialists, iterative design sustained by a software system, support to real case studies, and postdesign interviews (Figure 1). The evaluation of ESPIM software employed the heuristic evaluation method [39] (see Textbox SM1 in Multimedia Appendix 1), usability tests [40] (see Textbox SM2 in Multimedia Appendix 1), the Semantic Differential Scale [41] (see Textbox SM3 in Multimedia Appendix 1), and the User Experience Questionnaire (UEQ) [42] (see Textbox SM4 in Multimedia Appendix 1).

The methods used in this study were approved by the Brazilian Research Ethics Committee under case number 57875016.3.0000.5390. Study participation was voluntary and respected anonymity.

### Recruitment

Users were involved in the participatory design as specialists or target users. In the case of specialists, they form a convenience sample recruited via email at nearby research departments. Specialists took part as intervention planners; inclusion criteria were being a health or education professional and experience in planning and delivering interventions. Education specialists participated as educational issues are part of eHealth [43] and corresponding interventions influence, among others, socialization, cognitive support, and mental health [44-46].

Regarding the target users who participated in the case studies, specialists handled their recruitment as they were participants in their interventions.

The study also had the contribution of specialists in human–computer interaction (HCI). They were recruited via email from nearby research departments and software companies. Inclusion criteria were background in HCI and experience in conducting heuristic evaluations.

Professional designers, specialists in user interface (UI) and user experience (UX), were also recruited to design refined interfaces and validate them after the implementation. They were recruited via email from nearby software companies.

### Participatory Design

#### Evolution of ESPIM

Following a participatory design approach, the ESPIM method evolved according to requirements gathered from literature reviews, interviews, prototyping, and implementation of software instances of the associated model, and gradual usability evaluations (Figure 1). As observed by Byambasuren et al [47], usability is a main barrier for prescription and adoption of mHealth apps, especially for older people. To overcome these barriers, usability and UX were evaluated through empirical and inspection-based evaluations, which provided new requirements and detailed existing ones [48-51].

#### Interview (Predesign)

During requirement collection, specialists of different domains participated in semistructured interviews (Figure 1C and Multimedia Appendix 2) to discuss their needs in carrying out remote data collection and interventions, to answer a survey of the difficulties they faced in data collection and interventions, to present the system prototype as a potential solution, and to collect requirements. These professionals worked, among others, with children and adults with typical and atypical development, older people, pregnant women, and individuals with motor impairments. All participants conducted academic research in their areas. They were individually interviewed at their workplaces (by IZ and KRHR). Each meeting lasted 1 hour and 30 minutes on average. Interviews were performed with all participants who accepted the invitation.

#### First Heuristic Evaluation (App Prototype)

Four HCI specialists (Figure 1D), 2 researching usability for older people and 2 accessibility, conducted the first heuristic evaluation of the mobile interface of the ESPIM app prototype (question based at the time) [49]. The session lasted 24 hours so specialists could evaluate the 4 daily temporal triggers besides initiating the program themselves. The questions were related to daily routines and aimed to connect the trigger time with locations, daily activities, information technologies used, and ongoing activities.

#### First Usability Test (Web Application Prototype)

The first usability test performed on the web interface of ESPIM prototype (Figure 1F) aimed at answering: Do the specialists clearly understand what is the system and its purpose? Do the specialists face difficulties creating intervention programs using the system? Are the specialists able to complete all stages of creating intervention programs, including the setup phase? The test protocol (see Textbox SM5 in Multimedia Appendix 1) evaluated the understanding and the performance of the prototype from the point of view of health specialists using the metrics task execution time, number of steps to complete tasks, number of completed tasks, number of errors, and overall satisfaction.

We provided a hypothetical scenario *Geriatrician* outlining the tasks conducted by a specialist (geriatrician) who plans an intervention program to an older person with cognitive impairment who is attended by a caregiver (see Table SM1 in Multimedia Appendix 1). The scenario comprises 8 tasks requested of the older person, who is aided by the caregiver if needed. We specified the most complex task as a diagram (see Figure SM1 in Multimedia Appendix 1).

#### Checklist

The UI/UX designers who designed graphical interfaces executed a checklist-based evaluation (Figure 1J). Upon interacting with the system, they enlisted improvements.

#### Heuristic Evaluation

HCI specialists performed an inspection-based evaluation on the web application interface (Figure 1L1). The evaluators received an email with instructions, a task checklist, links, and files. The links led to the informed consent form, a profile survey, and to the ESPIM web application. The files contained instructions on how to conduct the evaluation, a template for reporting issues identified along with the corresponding heuristics, and the hypothetical scenario *Geriatrician*, comprising the tasks to be analyzed (see Table SM1 in Multimedia Appendix 1). After individual inspections, the evaluators met to discuss the problems found in the interface and produced a consolidation report.

The second heuristic evaluation of the mobile app followed the same protocol, using the same files (Figure 1J3). Additionally, the evaluators received the app installation file which included an intervention program and corresponding task checklist of the hypothetical scenario *Nutrition*. Four evaluators inspected the app performing 6 tasks: install, initiate and give the permissions requested by the ESPIM app, log in with your Google account, start the “Nutritional Data Collection” intervention program and navigate through all screens planned by the nutritionist, explore the app settings, and disconnect from the app.

#### Second Usability Test (Web Application) and Interview (App)

The second usability test on the web application (Figure 1L1) and the interview about usability and interaction aspects of the mobile app (Figure 1L2) were conducted at the same time. The questions and the protocol were those used in the first usability test.

For testing the web application, we provided the scenario “Monitoring and evaluating the performance of the older people in digital literacy courses through remotely programmed interventions” (see Table SM2 and Figure SM2 in Multimedia Appendix 1). Aspects of the web interface elements evaluated were ease of use, memorization, easiness to “undo” actions, learnability during use, intuitiveness, feedback/error messages, information organization, arrangement of interface elements, available features, and interface design. Participating specialists answered a 7-point Likert scale (1=“Awful” to 2=“Excellent”) for each aspect. The specialists also provided a self-evaluation of their performance using the system by responding to 7 affirmative sentences using a 5-point Likert scale (1=“Strongly agree” to 5=“Strongly disagree”): I easily completed the required tasks, I completed the tasks rapidly and efficiently, I would need someone’s support to use the system, I felt more productive during the interaction with the system, I easily found the information and functionalities that I needed, I needed to thoroughly think or remember before completing the tasks, and I would recommend the system to other people.

The specialists had previous experience as instructors or tutors in mobile digital literacy courses for elderly individuals (Figure 1H2) as part of the Case Study ElderlyDL (Table 1). They were interviewed regarding their learning while observing older people interacting with the app and commonly reported complaints. The questions asked were “Which errors or infrastructure problems were found? Which devices did not work? Which frustrations were observed while older people interacted with the app? For each type of task: What were the main difficulties of the older people? What were the issues? What could be better? What is good and should not change?.”

**Table 1:** Eight case studies.

Case study	Specialists involved	Participants	Short description
CS-ASDParents	2 psychologists; 1 computer scientist	3 families (3 children with autism spectrum disorder and 3 parents)	Promoting engagement in educational activities between children diagnosed with autism spectrum disorder and their parents.
CS-ElderlyDL	3 gerontologists, 2 computer scientists, 1 psychologist, 1 statistician	365 older people (age 60+)	Supporting mobile digital literacy courses for elderly.
CS-ClassTutors	1 gerontologist, 1 computer scientist	12 tutors (graduates/undergraduate students)	Analyzing elderly digital literacy courses using tutors’ feedback.
CS-MediaParcels	1 psychologist, 1 computer scientist	1 family (1 father and 2 children); 3 elderly friends (age 60+)	Encouraging multimedia interventions to promote social connection among elderly.
CS-SpeechTherapy	1 speech therapist	5 children (age 8-12)	Deploying speech therapy homework for children.
CS-OPCaregivers	1 occupational therapist	30 caregivers of older people	Providing informative contents for caregivers of elderly with dementia.
CS-OPStorytelling	2 computer scientists, 1 gerontologist	15 older people (age 60+)	Enabling the creation of digital storytelling by seniors.
CS-StoryReading	1 psychologist	45 children (age about 10)	Developing digital stories for children with reading disabilities.

#### Case Studies

##### Overview and Approval

The ESPIM method evolved supporting real case studies conducted by specialists (Figure 1) who used the method via the associated ESPIM system to manage interventions with their populations of interest [52,53]. These case studies are part of the empirical evaluations of the ESPIM system (Table 1).

Each case study was submitted and approved by the Brazilian ethics committee and all data were anonymized. Their common study protocol included the following: signing an informed consent form, filling out a pretest profile survey, filling out a posttest questionnaire about the interaction experience, and participating in a semistructured interview at the end of the study. Each specialist applied specific evaluation forms of their respective fields to analyze the results of their studies.

##### Case Study ASDParents

The case study ASDParents studied engagement in educational activities between children with autism spectrum disorder and their parents (Figure 1H1). Parents were instructed to conduct at least one out of three planned educational activities at home with their children, once a day, during the 6 weeks: the first 3 weeks used conventional paper-based written instructions, whereas the last 3 employed ESPIM. Psychologists employed ESPIM to send text and video tasks and to monitor task accomplishment and performance. All children studied at a Brazilian nongovernmental organization with a 2-hour/week workload. Three families (3 children and 3 parents) participated [54].

##### Case Study ElderlyDL

The case study ElderlyDL offered mobile digital literacy courses to older people (Figure 1H2). Computer science, psychology, and gerontology specialists employed the web application to design intervention programs as homework for the older people, who received and responded to the tasks via an app. During 13 weeks, the app sent, on weekdays, a notification around 7 pm alerting about the homework. The study involved 365 older people [48,50,55].

##### Case Study ClassTutors

Case study ClassTutors collected feedback from tutors assisting 3 instructors providing digital literacy courses for older people (Figure 1N1). The tutors were undergraduate and graduate students. One instructor (gerontologist) used the web application to guide tutors in evaluating the effectiveness of the classes and identifying situations of stress or struggle. The app sent 1 notification asking for feedback after the weekly class, the intervention being available throughout the week. A total of 12 tutors participated in this study for 4 months.

##### Case Study MediaParcels

In the Case study MediaParcels (Figure 1N2), 1 psychologist employed ESPIM as a multimedia exchange tool to investigate the impact of social interventions among older people and their connections. The specialist designed interventions to encourage participants to share self-revelations and media with affective content. The psychologist requested content from 1 participant, annotated the content with the meaning embedded in the original request, and forwarded it to the participant’s connections. One study involved family members (father and 2 children) and another study involved 3 elderly friends. Both studies lasted 2 weeks [56].

##### Case Study SpeechTherapy

The case study SpeechTherapy was applied in the clinical context of speech therapy (Figure 1N3). The specialists planned reading, writing, and comprehension tasks to complement activities conducted at the clinic. They applied remote interventions with 5 patients (aged 8-12 years) with reading or writing issues. Five children participated in the 5-month case study.

##### Case Study OPCaregivers

The case study OPCaregivers delivered information to caregivers of older persons with dementia toward guiding and qualifying the care provided (Figure 1N4). One occupational therapist created interventions to present information related to feeding, personal hygiene, guidelines for maintaining a structured and stimulating routine, and tips for managing behavioral symptoms. Thirty caregivers participated in this study.

##### Case Study OPStorytelling

The case study OPStorytelling employed ESPIM interventions to guide the creation of video stories by users with little experience in producing digital content, especially older people (Figure 1P1). One specialist used ESPIM to create interventions as “storytelling scripts” that combined requests for text, video, image, and audio assets. A dedicated service, integrated into the ESPIM software, received the media assets, generated the corresponding video, and uploaded it to a YouTube private channel. This study employed 2 workshops to teach 15 older people to produce video-based narratives [57].

##### Case Study StoryReading

In the case study StoryReading, a specialist in psychology used the ESPIM system as a tool to create instructional programs in the form of text-based stories, augmented with images and animations (Figure P21). The target users were children with reading difficulties. The goal was to improve reading comprehension by delivering stories integrated with questions and corresponding interactive feedback.

#### Interview (Postdesign)

A member of the ESPIM team (BCRC) interviewed the specialists after their studies (Figure 1R). The specialists responded to a semistructured 2-part interview: area of expertise and related studies, and how they modeled interventions in their studies. The latter aimed to elicit how specialists designed, delivered, and monitored interventions using the ESPIM system.

## Results

### User Statistics

Predesign interviews (Figure 1C) involved 12 health specialists: 5 psychologists (4 specialized in special education and 1 in behavioral psychotherapy), 3 nurses (1 specialized in public health, 1 in health sciences, and 1 in mental health), 2 physicians (1 specialized in obstetrics and gynecology and 1 in psychiatry, geronto-psychiatry, and neurology), 1 physiotherapist (observer in neuropediatrics), and 1 occupational therapist (specialized in public health).

The first heuristic evaluation of the mobile app (Figure 1D) was conducted by 4 HCI specialists (2 specialized in usability for the older people and 2 in accessibility).

The first web application usability test (Figure 1F) involved 5 psychologists (3 specialized in special education; 1 in science, technology, and society; and 1 in biology).

The checklist-based evaluation was realized by 2 UI/UX designers (Figure 1J1).

The heuristic evaluation of the web application (Figure 1J2) and the second heuristic evaluation of the mobile app (Figure 1J3) involved 4 HCI specialists: 1 inexperienced (never performed this kind of evaluation), 1 had intermediary experience (conducted 3 evaluations), and 2 were experienced (executed more than 3 evaluations).

The second usability test of the web application (Figure 1L1) and the interview about usability aspects of the app (Figure 1L2) were performed, in the same session, with 5 gerontologists and 1 occupational therapist.

Postdesign interviews (Figure 1Q) involved 8 specialists: 2 psychologists, 2 gerontologists, 1 occupational therapist, 1 speech therapist, and 2 computer scientists who offered digital literacy courses for older persons in collaboration with one of the gerontologists.

Target users who took part in the interventions via the ESPIM mobile app included 431 older persons, 30 caregivers of older persons, 5 children, 12 undergraduate/graduate students, and 3 families with children with autism spectrum disorder (Table 1).

### Participatory Design

#### Overview

The ESPIM comprises (1) a list of requirements for mHealth experience sampling and intervention-based methods and systems, (2) a 4-dimension planning framework, (3) a 7-step-based process for planning interventions, and (4) an ontology-based conceptual model. The ESPIM system and the cases study reports complement the contribution.

#### ESPIM’s Functional Requirements

The functions demanded from ESPIM were elicited using literature review, predesign interviews, iterative prototyping, and postdesign interviews. The resulting functional requirements (Table 2) concern creating and managing (1) the “intervention programs,” (2) the “persons” involved as observers and participants, (3) the “events” constituting the program and comprising triggers and tasks, (4) the “active tasks” specialists request to target users, (5) the use of “sensors” to capture data or trigger tasks, or both, and (6) the access to the “results.”

**Table 2:** Functional requirements (FRs) for ESPIM.

FR		Description
**Intervention program**		
	FR01	Enable creation, management, and reuse of intervention programs.
	FR02	Enable definition of open or fixed beginning and ending dates for programs.
	FR03	Enable organization of programs into phases composed by different events.
**Person**		
	FR04	Enable registration and management of observers.
	FR05	Enable registration and management of participants.
	FR06	Enable collaborative management of programs.
	FR07	Provide user authentication/authorization with roles and permissions.
	FR08	Enable multiple associations among participants and programs.
	FR09	Enable creation of contact lists related to privacy control.
	FR10	Enable the association of relationships among participants (eg, communication).
	FR11	Enable importing participants’ data from external sources.
**Event**		
	FR12	Enable creation of active tasks and sensor-based sampling.
	FR13	Enable the association of triggers to events.
	FR14	Enable configuration of intrusiveness level in triggers.
	FR15	Provide trigger types: self- and specialist-initiated, temporal, contextual, random.
	FR16	Enable definition of triggers’ timeout.
	FR17	Enable color-coding events.
	FR18	Enable annotations and follow up via participant’s app interface.
	FR19	Enable configuration of triggers disabling when a condition is fulfilled.
	FR20	Enable configuration of alert to inform when a participant did not answer a trigger.
	FR21	Enable configuration of alert to inform when a participant answered a trigger.
	FR22	Enable configuration of triggers rescheduling when a condition is satisfied.
	FR23	Enable configuration of alert to inform of a specific answer (eg, risky behavior).
	FR24	Enable configuration of automatic processing of responses (eg, condition based).
**Active tasks**		
	FR25	Provide active tasks type message, question, media request, and external app launch.
	FR26	Enable active tasks containing multimedia stimuli and emphases-enriched text.
	FR27	Provide open-ended, multiple/single-choice (including pictures as choices) questions and scales (eg, Likert, sorting scale, grid).
	FR28	Enable definition of mandatory active tasks.
	FR29	Enable configuration of interaction flows (skip, branch, and loop).
**Sensor-based sampling**		
	FR30	Enable configuration of sensor-based sampling while interacting with active tasks.
	FR31	Enable definition of time intervals for sensor-based sampling.
	FR32	Enable configuration of automated sensor-based sampling.
	FR33	Enable sampling from software- or hardware-based sensors (eg, mobile and wearable devices, accessories, home sensors).
**Results**		
	FR34	Provide different filter modes for results’ visualization.
	FR35	Provide date, time, and duration of the corresponding result.
	FR36	Enable download of the results.
	FR37	Enable configuration of automatic analysis of responses by dedicated algorithms.

#### ESPIM 4-Dimension Planning Framework

**Figure 2:**
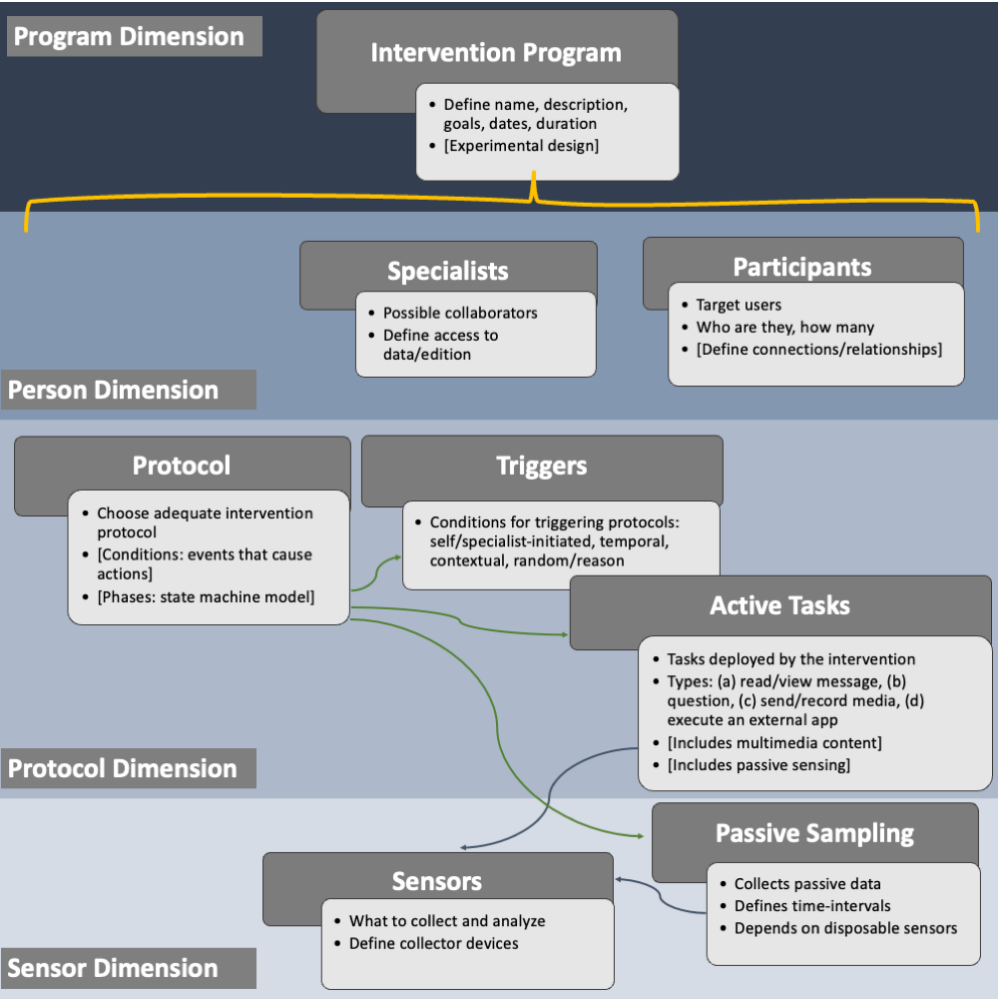
ESPIM Intervention dimensions: Program, Person, Event and Sensor.

Defined upon the requirements, an ESPIM intervention program involves 4 dimensions: program, person, event, and sensor (Figure 2). The *program* dimension comprises the intervention program as a whole, and characterizes general intervention settings, such as name, definition, description, goals, duration, and, in research-based situations, its experimental design.

The *person* dimension comprises the specialists in charge of the program and their target users, also called participants. Both professionals and researchers may work with collaborators who require distinct access control levels. Moreover, establishing target users is a key aspect of an intervention. In analytical work, the number of participants and their characterization are crucial, while individualized interventions and use case scenarios may demand the participation of other individuals (eg, parents, caretakers, partners). Relationships between participants and these connections, along with delimited data sharing, might be considered in the program.

The *protocol* dimension comprises features provided by mobile technologies, aggregating the flow(s) that constitute the intervention along with the corresponding time- or sensor-based triggers. Finally, the *sensor* dimension allows expressing sensor-based support both for data collection and for triggering events.

#### ESPIM 7-Step-Based Process

##### Overview

**Figure 3:**
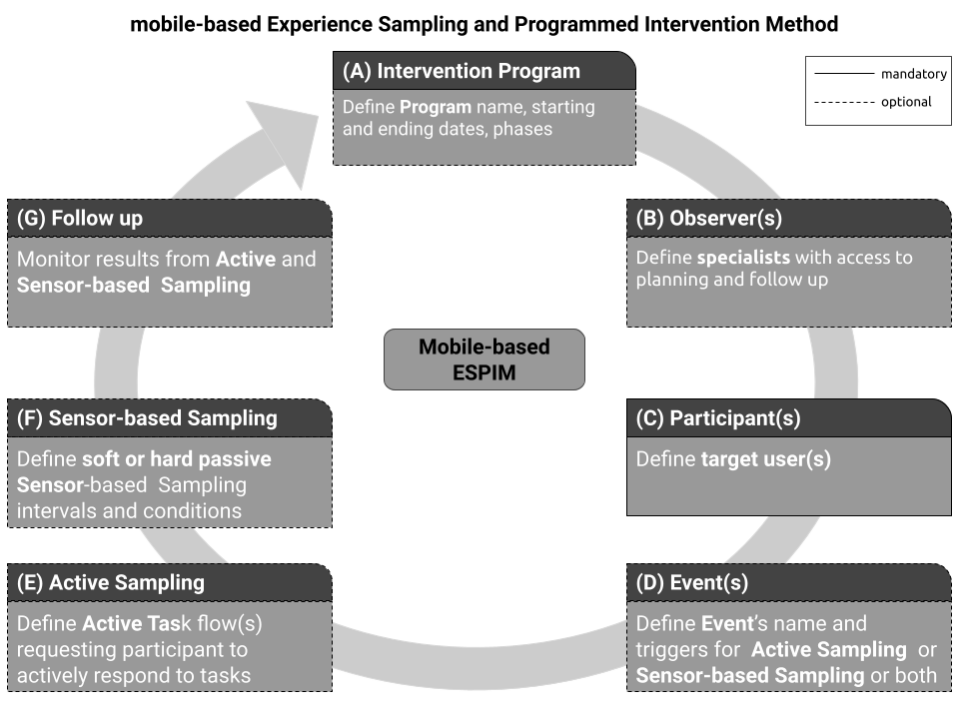
ESPIM 7-step process.

ESPIM directs specialists in iteratively planning a mobile-based intervention program (Figure 3). The 7-step-based process suggests first identifying the intervention program by a name and defining its overall duration (Figure 3A), followed by the identification of who will have access to the planning procedures (Figure 3B) and who the target users are (Figure 3C). The intervention program is then defined in terms of 1 or more events (Figure 3D) which combine triggers to sampling procedures formulated as active tasks (Figure 3E) or sensor-based sampling (Figure 3F). Once the intervention program is deployed, specialists monitor participant’s interaction and data collected (Figure 3G). These procedures are detailed next along with the corresponding requirements (Table 2).

##### Intervention Program: Name and Duration

The first step (Figure 3A) is to identify the intervention program by a name so that specialists can refer to the program for deployment, management, reuse, and change (FR01 in Table 2).

At the time of design, the intervention program deployment dates may not be determined. A program may be designed to be reused at distinct times, with different participants. Thus, when the intervention program is devised, the definition of starting or ending dates is optional (FR02).

An intervention program may be designed and structured to be applied in separate stages or phases delimited by time or other completion conditions (FR03).

##### Observers: Persons Who Have Access to the Planning Procedures

The second step is to register other specialists with access to the program (FR04 and FR06). This enables cases in which an intervention program is collaboratively designed or deployed, or both, by more than 1 specialist. Specialists that are responsible to manage intervention programs are called observers, and the optional step “observers” (Figure 3B) is employed when collaborators other than the one creating the intervention program should have access to its specification and results.

##### Participants: Persons Who Receive the Intervention Tasks via Their Mobile Device

The third step (Figure 3C) is to register the target users (participants) of the program (FR05). However, the actual target users may not have been determined when the intervention program is initially designed. Moreover, an intervention program may be designed to be reused with different participants at distinct times. Thus, when first creating the intervention program, the observer may not include real target users. Clearly, defining a participant is mandatory for deploying an intervention program. One strategy adopted by specialists in the case studies we report was to include themselves as participants, allowing them to test the intervention program before deployment by assuming a participant role (FR07).

Observers may include 1 or more target users in 1 or more programs (FR08). This characteristic is essential to provide flexibility to personalize programs for individualized monitoring or to monitor groups of target users. Specialists register participants’ contact information and aliases targeting users’ privacy (FR09).

Another type of participant represents persons having relationships to target participants, such as family members or caretakers, with whom specialists may interact. Thus, a requirement is support for relationship among participants and associating different events with distinct participant roles in the relationship, which should be personalized according to each case (FR10).

Finally, specialists may use existing information systems to import participants’ data valuable to intervention planning. Therefore, a service-based approach should be considered for third-party system integration and information exchange (FR11).

##### Event(s): What Sets of Tasks Are Triggered, and When, in the Participant’s Smartphone

An intervention program consists of 1 or more intervention events executed along a time frame. Each event (Figure 3D) is identified by a name which is used for reference within the intervention program itself or in other programs, warranting reuse of events in particular, additionally to the reuse of programs (FR01).

To each event, the observer associates a set of tasks to be put into effect in the participant’s mobile device. The actual tasks are defined by means of an active set (Figure 3E) or via sensor-based sampling (Figure 3F), or both (FR12). In the first case, participants respond explicitly to a task, for instance, by answering a question or capturing a video. In the second case, data are gathered passively from the participant’s smartphone, for instance, via sensors or automated logging routines.

Further, to each event the observer associates 1 or more triggers specifying the times in which event’s tasks are to be executed in the participant’s mobile device (FR13). This implies that, at the times planned by the specialists, the participant’s smartphone receives a notification corresponding to the event. If the notification triggers a flow of active tasks (ie, intervention flow), a notification is presented in the participant’s device at the level of intrusiveness specified (FR14); when the participant responds to the notification, the mobile app allows the participant to interact with the intervention flow. In cases in which the notification triggers a sensor-based sampling routine, the corresponding sensor or automated routine is executed.

Triggers should be of diverse types besides being time based (FR15) and should be associated with a timeout (FR16). Specialist-initiated triggers allow observers to launch tasks on their own initiative. Self-initiated triggers are necessary when specialists opt to allow users to execute an event at their own initiative by starting the mobile app themselves at any time. A random trigger is appropriated when specialists demand the event to start at unconventional times without a predetermined pattern. A contextual trigger is set off when a particular situation occurs, such as one defined by rules involving 1 or more conditions associated with physical sensors (eg, global positioning system coordinates or heart rate monitor) or software-based data (eg, agenda). In any case, the specialist may indicate that different events are of distinct types by using coding such as different colors (FR17). Moreover, an event that has been already completed may be indicated as such in the mobile app, along with other information that allows participants to be aware of their status in the intervention (FR18).

Furthermore, how a participant reacts to a trigger may be an important aspect to some studies; for instance, a trigger may need to be rescheduled or the observers should receive an alert when a participant did not answer (FR19-FR24). Contextual triggers (FR15) and automatic responses processing (FR24) are essential requirements if specialists consider ecological assessment for adaptive interventions (EMA and JITAI).

##### Intervention(s): What Flow of Tasks Are Explicitly Demanded via Participants’ Devices

An intervention flow (ie, active set; Figure 3E) is specified by the observer (FR12) to be presented to the participant via a mobile app in a customizable flow of active tasks (FR25). An active task is an intervention-based component that may contain 1 or more multimedia stimuli allowing, among others, sending instructions and requesting information; text-based stimuli should support emphasis including bold and italics (FR26). A question is a type of active task which comprises different formats (FR27) and they may be mandatory or not (FR28). One type of question, single-choice question, allows associating a different flow with each of the alternatives defined, as a result, conditional parallel flows (FR29). An active task may interact with a third-party mobile app, for both activating that app with customized configuration and collecting data resulting from its execution (FR25). An active set may also trigger a passive collection of sensor data without the need for an explicit user intervention (eg, capturing the face expression during the task; FR30).

##### Passive Sampling: What Information Will Be Collected Without Explicitly Asking the User

Passive sampling is needed when the design of the intervention program makes use of passive data collection without interrupting the participant (Figure 3F). This can be initiated via the configuration of temporal intervals (FR31) or of automated sensor or software-based data collection (FR32), or via association with an active set (FR30). The collection may use sensors and software executing in devices other than the participant’s smartphone such as wearable devices, accessories, and home sensors (FR33).

Passive sampling may be executed without an associated active set, as illustrated by Harari et al [58] when collecting data from sensors and logs. When this is the case, the observer specifies the sensors to be used as well as the conditions and the intervals of the collection.

##### Results: When and How Participants Participated in the Intervention Program?

In some interventions, specialists demand monitoring how participants engage in the program to measure its impact or to adjust the intervention according to users’ behavior and responses, or both (FR34 to FR37). When this is the case, observers need access to follow-up components which give access to results (Figure 3G). Another type is that in which specialists design an intervention program to provide information or to send instructions to participants, as is the case with tutorial apps or self-care apps [59]. When this is the case, the specialists do not need information on how and when users interacted with the program, which demands the follow-up procedure to be optional.

#### ESPIM: Ontology-Based Conceptual Model

**Figure 4:**
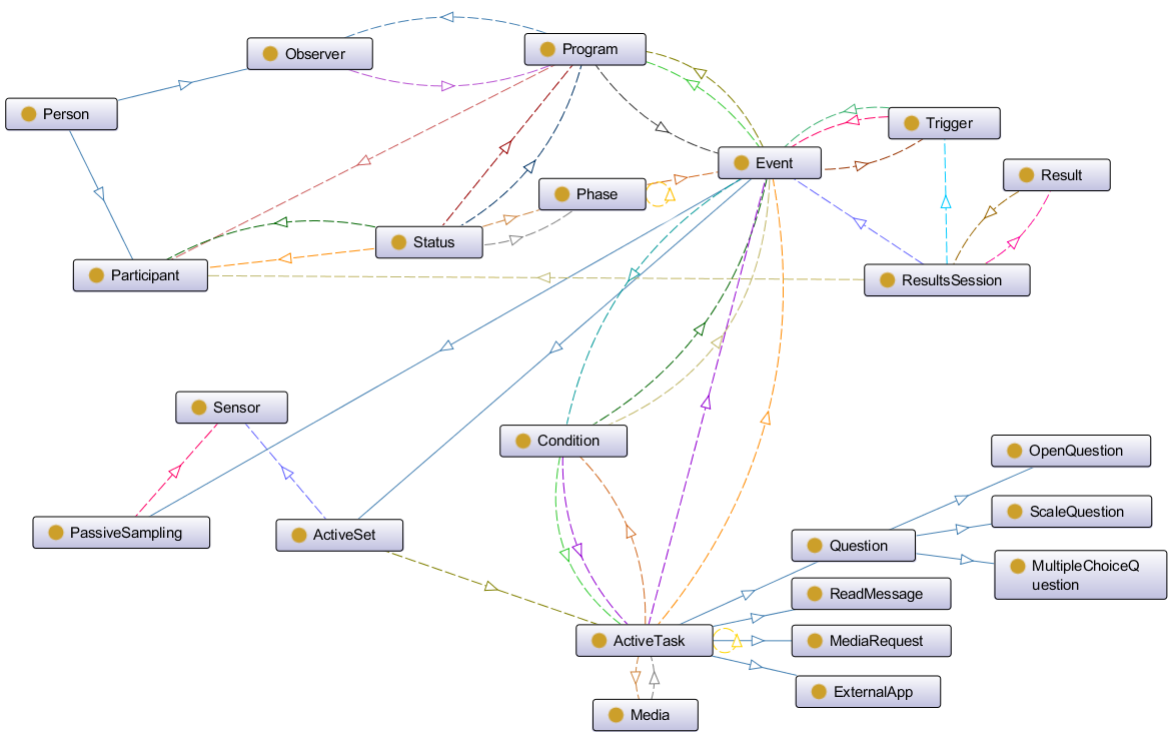
ESPIM: Ontology-based Conceptual Model.

Our study contributes to a conceptual model (Figure 4) guiding specialists in adopting existing software platforms or building new ones along with a software development team. We represent the conceptual model using an ontology given its wide adoption [60-64] and description power [65].

In the ESPIM ontology, the *Person* class represents observers (specialists) and participants (target users). The person concept may retrieve data from existing information systems using a unique key (eg, email). Participants may have relationships with 1 or more users in the system (eg, child, caregiver, partner).

The *Program* class represents an intervention program that encompasses a set of events that defines sets of active tasks or sensor data sampling (sensor-based sampling). Observers manage programs individually or collaboratively.

The *Event* class represents personalized or standardized intervention events applied by observers. An event comprises sets of tasks (active set) or sensor-based sampling. The *Active Set* class represents tasks required explicitly for a participant, represented by classes contained in the *Active Task* class. The *Sensor* class represents tasks achieved via sensor-based sampling, which may demand continuous and unobtrusive data collection at defined time intervals. An active set can be associated with sensor-based sampling occurring while the participant performs the tasks. Events initiate according to defined trigger conditions (in *Trigger* class).

The *Trigger* class encompasses trigger conditions and an overall set up that determines when and how an event is triggered. A trigger can hold the following features: self-initiated, temporal, contextual, random/reason, and specialist-initiated trigger. A self-initiated trigger allows target users to start an event, a temporal trigger schedules events based on time and dates, a contextual-based trigger considers context information obtained by sensors or by device usage, and random/reason-based triggers randomly deliver a defined number of intervention events during a period. Observers may control triggers remotely. A trigger setup indicates notification timeout and obtrusiveness level.

The *Active Task* class corresponds to the delivery of stimulus that requires an interactive response. An active task contains at least one media stimulus (eg, text, image, audio, video). In the model, an active task comprises 4 types of stimuli: read message, question, media request, and external app.

In the *Active Task* class, the *Read Message* class represents sending multimedia messages to users. The *Question Active Task* class represents questions in different formats, including open-ended, multiple- and single-choice, and scales (eg, Likert). A question may include textual or other media elements. Choice questions represent loops and branches. A *Media Request Active Task* class represents the request for media assets (eg, audio). Finally, the *External App Active Task* class represents the activation of external apps, sending customized activation values and receiving completion results.

The *Media* class represents media stimuli employed in an active task instance. The model admits adding multiple media elements to a task.

The *Sensor* class represents the set up associated with sensor-based sampling: what should be collected (eg, social interaction, facial expressions) by which device (ie, wearable devices). Collection may occur over a period (sensor-based sampling) or during user interaction (active task).

The *Result* class represents data collected by the intervention program via active tasks or sensor-based sampling. Moreover, every participant interaction, or lack of interaction when expected, should be logged. Observers access instances of the *Results Session* class.

The *Condition* class represents actions triggered by conditional rules encompassing active (user interaction based) or passive (sensor based) data. For instance, if a participant fails to answer a notification, it is possible to execute an action such as alerting a particular observer or scheduling a new trigger.

The *Phase* class represents the organization of an intervention program in stages. The *Phase* class aggregates events, and each event may be included in 1 or more phases. A condition that defines when a participant should proceed to another phase defines a phase duration. Conditions may be time, contextual, or response dependent. Moreover, the *Status* class in the ESPIM ontology allows registering a participant’s progress in an intervention program that has phases.

#### ESPIM Software

**Figure 5:**
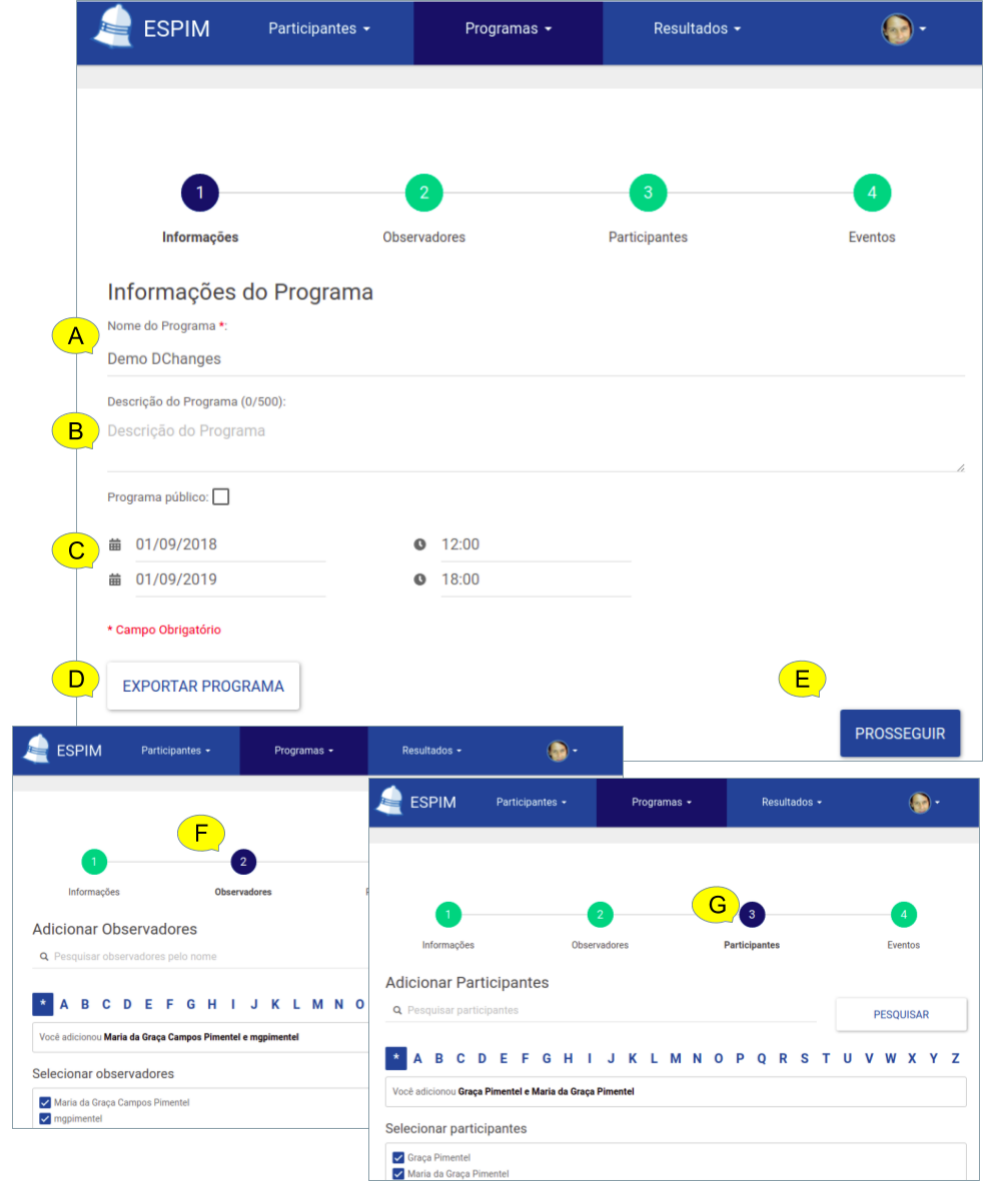
ESPIM web application used by the specialist to create an Intervention Program. The first step (A) informs the program’s name and description (B), and duration (C). Options include exporting the program (D). The following steps register specialists (observers-F) and target-users (participants-G).

**Figure 6:**
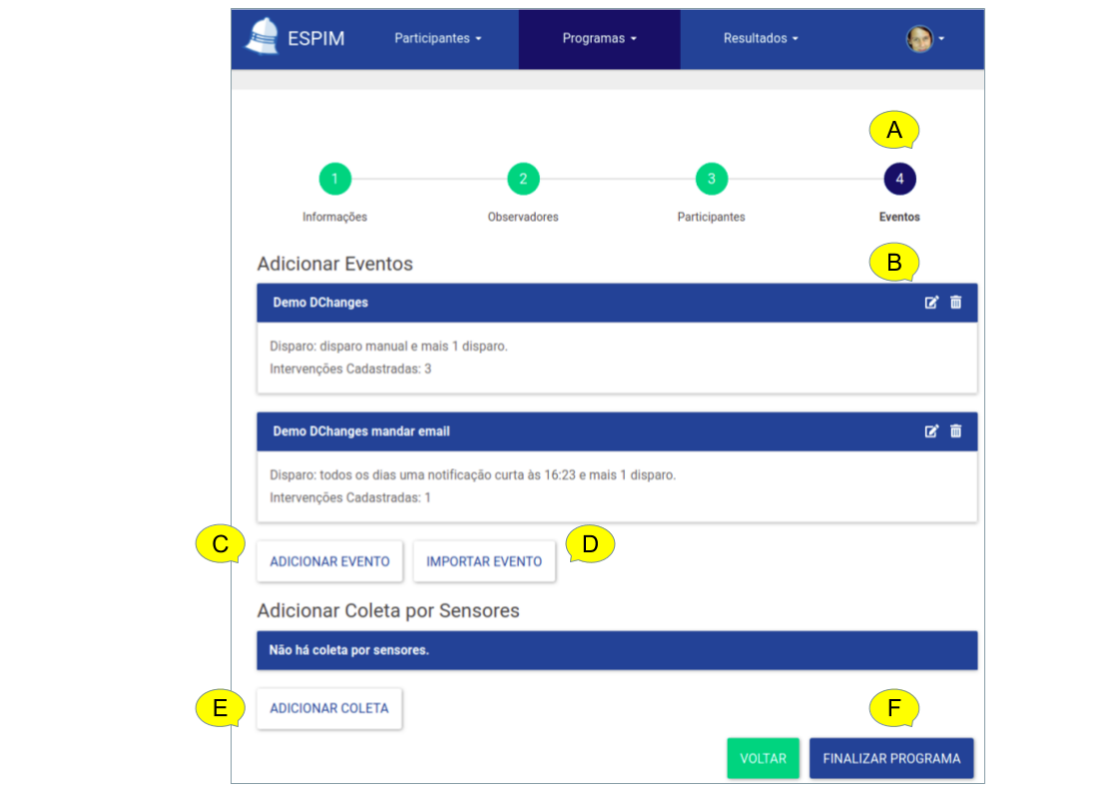
ESPIM web application step with options for Events. Options in this step (A) include editing an existing event (B), editing a new event from scratch (C), or by importing an existing one (D), specifying collection based on sensors (E). Finalizing is always available (F).

**Figure 7:**
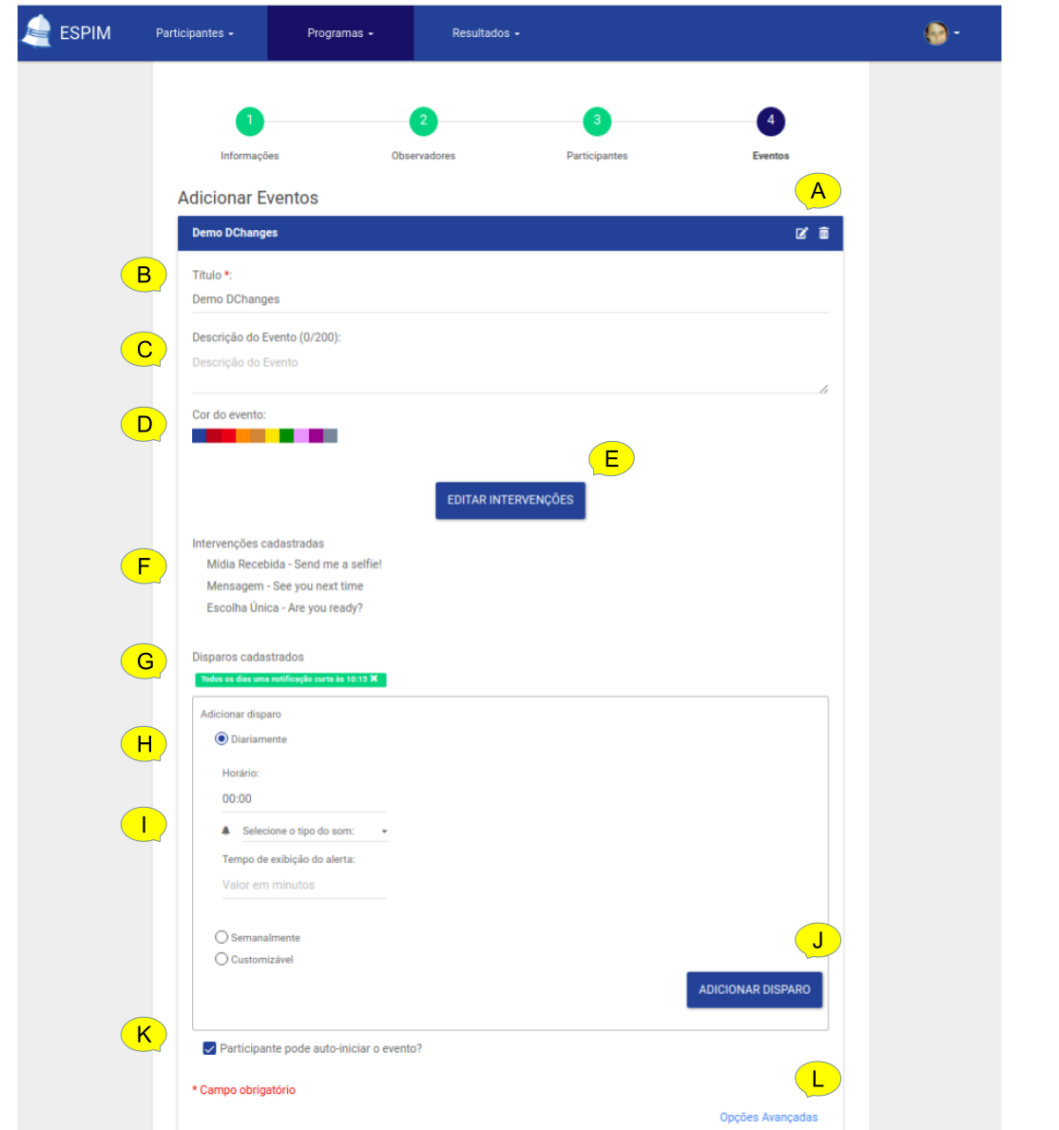
ESPIM web application step for editing one Event. For each Event (A), specialists provide a name (B), description (C), and color-coding (D). A button gives access to the interface for editing the corresponding flow of active tasks (E). This step shows the text from existing interventions (F) along with current triggers (G). Specialists create time-based triggers (H), and configure (I) and save the corresponding alarm types. Specialists can set self-initiated events (K) and configure that observers receive alerts when participants interact or miss an alarm (L).

**Figure 8:**
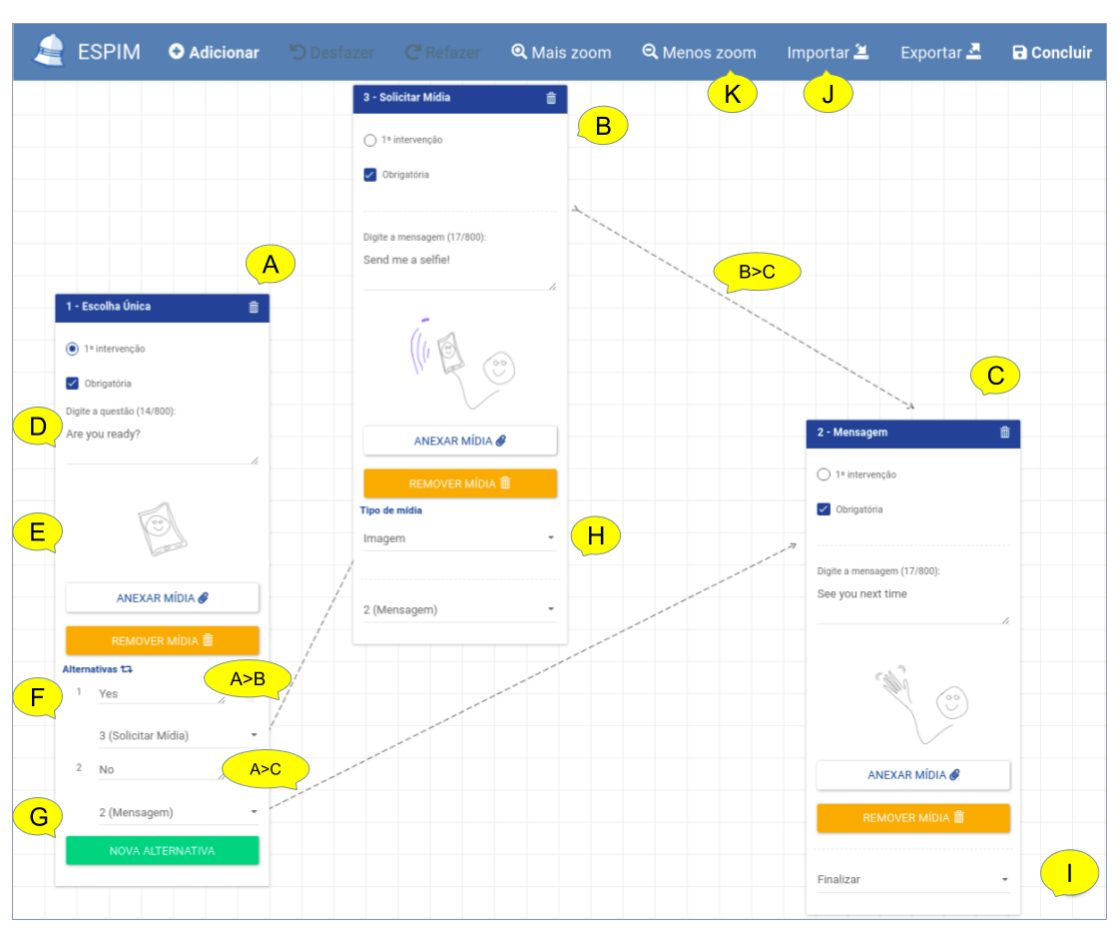
ESPIM web interface to create an Active Task Flow. This flow contains three interventions: a single-choice question (A), a task-based intervention requesting an image (B), and a message intervention (C). Specialists may include instructions using text (D) or other media (image (E), audio or video), or both. They must indicate the initial intervention (radio button in A), and mark each intervention as mandatory or optional (checkbox in A-C). The app shows arrows to indicate the flow (eg B>C). In a single-choice intervention (F), specialists may associate specific interventions to each alternative (A>B and A>C). Also, they can choose among many alternatives to choice- and scale-based questions (G). When specialists create a task requesting media (B), they indicate the type of media required (“image” in H). They must nominate at least one closing intervention (I). The specialist can zoom (K), and import and export (J) flows.

**Figure 9:**
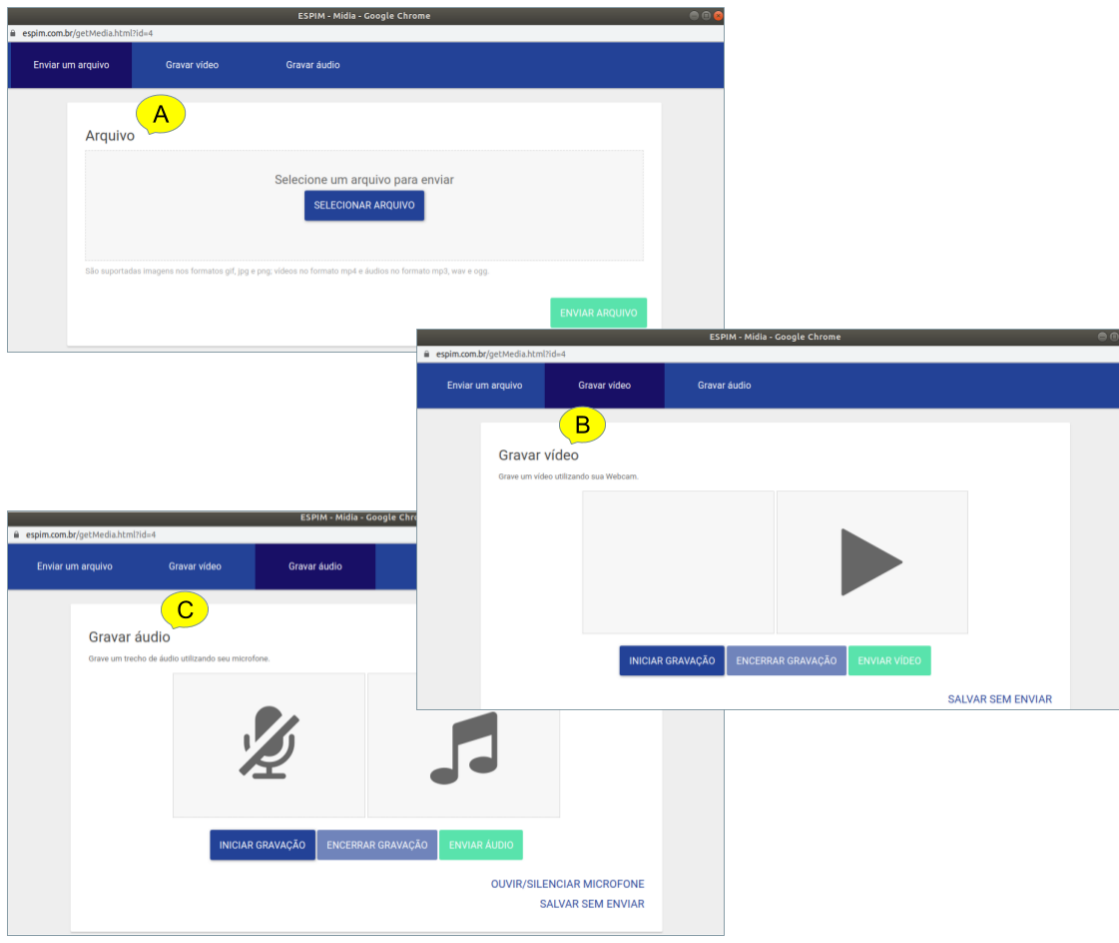
ESPIM web interface to upload media-based stimuli (A) and to record video (B) or audio (C).

**Figure 10:**
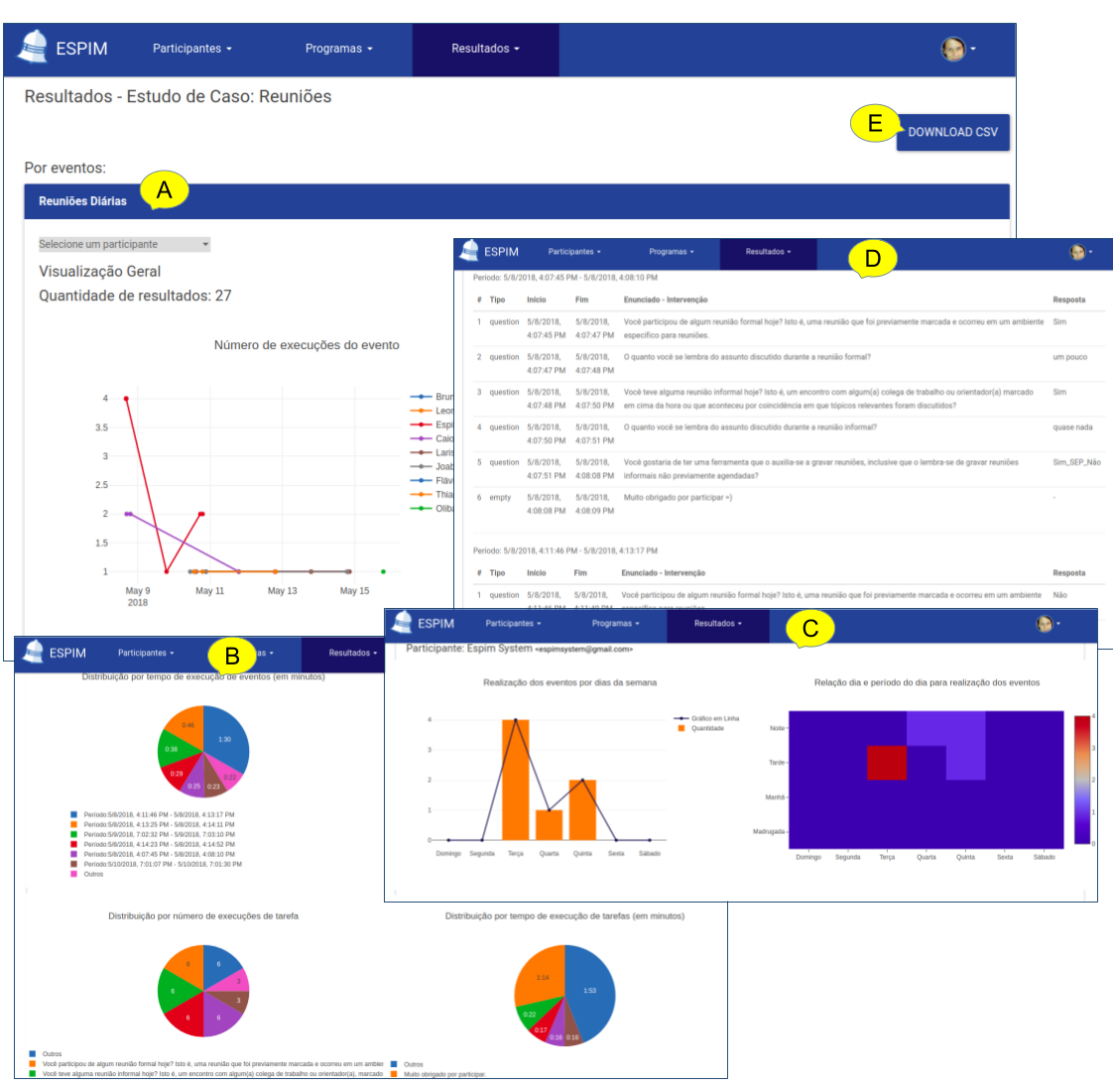
ESPIM web interface to visualize results includes an overview by a participant (A), distributions of responses both per task (B) and along the time (C), access to individual responses (D), and an option for download (E).

**Figure 11:**
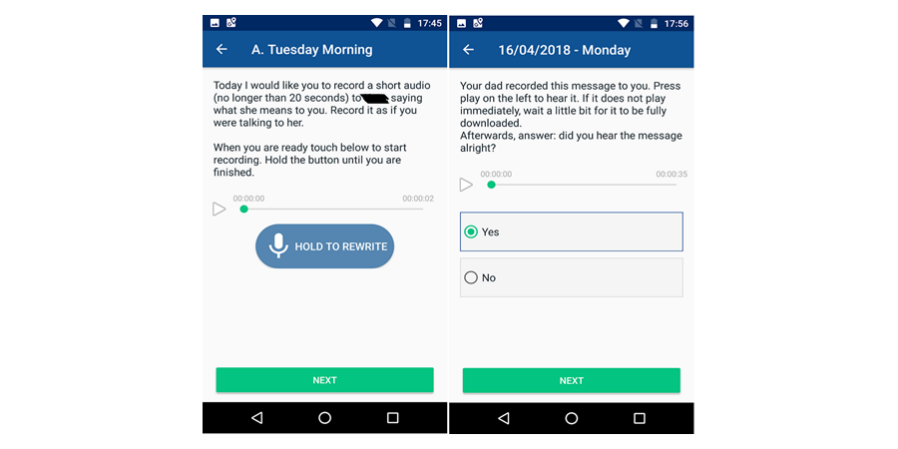
Mobile app presenting interventions. Active task requesting the user to record an audio message (left). Active task inviting the user to respond to a single-choice question upon listening to an audio stimulus (right).

The iterative design leading to the ESPIM method (Figure 1) involved the iterative prototyping of the software instance (employed by specialists when authoring; Figures 5-8) and monitoring (Figures 9 and 10) an intervention program, and the mobile app used by participants (Figure 11; see Multimedia Appendix 3 for details).

### Evaluation Outcomes

#### Interview (Predesign)

The predesign interviews lead to eliciting requirements from a group of specialists from several backgrounds (Figure 1C). We consolidate the results into ESPIM’s functional requirements (Table 2).

#### First Heuristic Evaluation (App Prototype)

For the app prototype (Figure 1D), the HCI evaluation team reported the following issues: (1) navigation problems (store navigation and branching paths, confirm before quitting, clear paths upon abandonment/finishing); (2) inconsistent display on different versions of the operating system; (3) improve screen use (landscape orientation and fix overlay in small screens when the virtual keyboard is visible); (4) generic/uninformative notifications; and (5) nonstandard fonts. We fixed these issues and applied the material design guidelines [66].

#### First Usability Test (Web Application Prototype)

Each specialist interacted individually with the web application (Figure 1F) according to an 8-task hypothetical scenario (see Table SM1 in Multimedia Appendix 1). ESPIM researchers (including KRHR and IZ) observed the interaction. Upon conclusion, specialists answered the evaluation questionnaires and participated in a semistructured interview to report problems, feelings, and expectations. Results from each task are as follows.

Specialist P1 could not complete tasks 7 and 8 as she could not find the “Save” button. Specialists P3 and P5 partially completed task 7 (see Figure SM1 in Multimedia Appendix 1) as they added extra activities to the intervention planned. Regarding errors, specialists P2, P3, and P5 made 4 errors; P4 and P1 made 6 and 9 errors, respectively. The errors included creating an existing observer or participant, opening an external page during the interaction, using the first intervention element (a star icon) as a “Save” button, trying to save an empty program, and unexpected interactions with the interventions’ flow screen.

Regarding the tasks, specialists made more than 1 error in tasks 4 and 7. In task 4, all specialists were uncertain about the meaning and function of the “observers” role. Task 7 was the one in which the specialists presented more difficulty and made more mistakes. This task had several substeps and demanded more attention, so specialists spent the longest (mean 13 min [SD 0.55]).

Results from the subjective evaluation of the interface elements range from –3 to +3. Specialists rated positively most aspects evaluated. Aspects evaluated +2 (Good) and +3 (Excellent) were available features, ease of memorization, and learning during use. The items with the lowest ratings were feedback/error messages, ease to undo errors, and information organization of the system. One negative score (–1) was attributed to “ease to undo errors” by P1 and was consistent with her overall performance. P1 could not fulfill 1 task and partially fulfilled another (tasks 8 and 7, respectively). P1 also made the highest number of errors.

The results from the UX while interacting with the system were positive, ranging between 1.4 and 3 (–3 to +3 range), meaning that specialists associated their experience with positive qualifiers, concepts, or feelings. The most positive evaluations were “efficiency” (mean 2.55 [SD 0.60]) and “dependability” (mean 2.35 [SD 0.87]). Evaluation of the remaining factors were as follows: attractiveness, mean 2.16 (SD 0.79); perspicuity, mean 2.10 (SD 0.78); stimulation, mean 2.10 (SD 0.71); and novelty, mean 2.05 (SD 0.68). As a result, specialists considered that they could interact efficiently, execute tasks reliably, understand and find functionalities, and felt stimulated and motivated during the interaction. They also perceived the system as innovative and attractive regarding interface elements.

#### Checklist

The UI/UX designers who designed the graphical interfaces of the ESPIM web applications suggested 9 improvements to the corresponding implementation (Figure 1J), including components behavior according to the material design guidelines, user lists presentation order, and text size and spacing. We adjusted them accordingly.

#### Heuristic Evaluation

For the heuristic evaluation of the ESPIM web application (Figure 1J2), the evaluators’ report contained 71 usability issues. They classified 24 problems with severity 1 (cosmetic), 16 with severity 2 (minor), 26 with severity 3 (major), and 5 with severity 4 (catastrophic). Evaluators associated problems with 4 heuristics: consistency and standards, user control and freedom, flexibility and efficiency of use, and aesthetic and minimalist design. The ESPIM team promptly solved issues classified with severity 3 and 4 to provide a version for tests with gerontologists (Figure 1L1 and Figure 1L2). We fixed the other problems in later versions [52,67].

For the heuristic evaluation of the ESPIM mobile app (Figure 1J3), the evaluators’ report comprised 21 usability issues. They classified 4 problems with severity 1 (cosmetic), 6 with severity 2 (minor), 8 with severity 3 (major), and 2 with severity 4 (catastrophic). Although evaluators associated 1 issue with the user control and freedom heuristic (7/21), we argued that we purposely designed some guiding-based features to make the app accessible to a wide range of user profiles. Yet, evaluators considered that several icons and some nomenclature lack intuitiveness and associated the issues with the correspondence between the system and the real-world heuristic (5/21). Evaluators associated other issues with the following heuristics: system status visibility, recognition instead of memorization, flexibility and efficiency of use, and aesthetic and minimalist design. We solved the problems before tests with gerontologists (Figure 1L1 and Figure 1L2).

#### Second Usability Test (Web Application) and Interview (App)

For the second usability test of the web application (Figure 1L1), each specialist interacted with the system according to a set of 10 tasks contemplated by 1 hypothetical scenario (see Table SM2 in Multimedia Appendix 1). ESPIM researchers (including KRHR and BCRC) observed the interaction. According to the protocol, the specialists answered the evaluation questionnaires and participated in a semistructured interview (see Textbox SM5 in Multimedia Appendix 1).

Specialists P1, P4, P6, and P5 completed all tasks. Specialists P2 and P3 partially completed task 7 (see Figure SM1 in Multimedia Appendix 1) because they did not find the requested media for uploading in the computer used for the test. Regarding errors, most happened at the planning interventions screen (Figure 7). Some specialists (3/6) faced difficulties in defining the intervention flow, especially the beginning and end.

Regarding the tasks, specialists made more than 1 error in tasks 4, 7, and 8. In task 4 (creating and adding a new observer), the specialists had difficulties registering emails in the interface. Task 7 was again the one in which the specialists made more mistakes. They spent more time executing task 7 (planning intervention flow; mean 15 min [SD 0.7]) as this task required more attention and the most steps. In task 8 (adding triggers), specialists faced difficulties understanding interface elements. For all other activities, the average execution time of participants was less than 2 min (SD 0.43).

Results from the questionnaire reporting subjective evaluation of interface elements were positive in most aspects. Aspects evaluated between “good” and “excellent” were layout of interface elements, available features, and ease of memorization. Five of six evaluators rated the aspect “learning during the use” between “excellent” and “very good.” Specialist P2, who did not complete the tasks, evaluated this aspect as “too bad,” reported “I really liked using ESPIM, it seems to me a very pleasant, intuitive tool” and offered the following suggestion, which we incorporated later: “[...] sometimes I lost a description explaining what the specific term meant: having short and clear information would have facilitated my journey.”

At the concluding interviews, the specialists provided input as instructors or tutors in digital literacy courses for older people (CS-ElderlyDL; Figure 1H2). Specialists remarked as positive the simple aesthetic of the interface with “well-chosen colors” (colors considered color-blind users) and the in-app back button, justifying that some older people face difficulties finding the smartphone back button. Interviewees related that the app improved the engagement and learnability of the older people in the classes (especially when compared with control groups), multimedia messages promoted positive emotions, and mobile-delivered homework helped avoid evasion. Interviewees also reported some issues: users did not understand the asterisk in mandatory fields (consider disabling the next button), users did not know how to react to open questions (consider the automatic display of keyboard), users did not notice that a media was already captured (consider feedback with media preview), users clicked outside of multiple-choice options (consider exhibiting them inside inline boxes), lack of configuration for notification intrusiveness, and lack of in-app configuration for enabling/disabling 3G network usage.

#### Interview (Postdesign)

We conducted semistructured interviews with 8 specialists to identify the limitations they faced while conducting their case studies (Figure 1R). We classified the limitations as related to the model or the software. Model-related limitations imply modifying or extending the model to allow representing the required solution. Implementation-related limitations are restricted by the current system version: the model represents the corresponding solutions, but these are not yet available in the system. Because the latter are implementation specific, we discuss only model-related limitations.

Interviewees reported 4 limitations that require revisions in the conceptual model. The first limitation is related to the lack of support to represent the management of participants’ records. Such a support would allow specialists to register information they consider relevant. To allow representing such a feature, we must extend the model accordingly.

The second limitation concerns representing the organization of participants into groups other than direct relationships among participants (as per FR10). The solution requires creating a class that allows multiple associations between specialists, participants, and groups so that a participant may be part of various groups managed by different specialists.

The other 2 limitations concern groups of events: we should extend the model to represent both hierarchical and customizable groups of events. The analogous implementation would allow the corresponding visual customization of groups of events.

Although these limitations require changes in the conceptual model, they did not limit the planning, authoring, or deploying of the interventions by the specialists.

## Discussion

### Principal Findings

Our study tackles limitations faced by health professionals determined to conduct mHealth interventions. Our main results are the ESPIM method and the conceptual model, which succeeded in leading specialists in planning, authoring, and deploying mHealth intervention programs with the support of a representative software system. ESPIM comprises (1) a list of requirements for mHealth experience sampling and intervention-based methods and systems, (2) a 4-dimension planning framework, (3) a 7-step-based process, and (4) an ontology-based conceptual model. A subsidiary result is the ESPIM software.

The list of requirements, offered to specialists when planning and deploying interventions, results from literature review, predesign interviews, iterative prototyping, and postdesign interviews. The 4-dimension planning framework aims to guide the planning of mHealth interventions by specialists by clarifying elements to be considered when using mobile, wearable, and ubiquitous technologies. The framework helps designing an intervention program while supporting team communication. The 7-step-based process guides the procedures that support ESPIM-based software.

A main contribution of ESPIM is a conceptual model aimed at guiding specialists in adopting existing software platforms or building new ones. The ESPIM ontology–based conceptual model represents the requirements collected and refined during our long-term design experience with health specialists and their research/professional needs. As a conceptual model, it defines a process and a common vocabulary to plan mobile device–mediated interventions; it also aids the development of mHealth apps.

Overall, ESPIM components can guide the planning and deployment of an intervention program using existing solutions, drive selecting one among the available tools, or guide the implementation of a novel specific or general platform.

While inspection-based usability evaluations of the ESPIM interfaces identified problems, we used the HCI specialists’ feedback to make continuous improvements to the ESPIM system. Such evaluations preceded usability tests with professionals and their target users, and the resulting adjustments lead to intuitive and satisfactory usability tests.

### Limitations

Limitations of our study are associated with those of the contributions. With respect to the list of requirements, even though requirements were elicited via interviews, iterative design, and empirical evaluation, some requirements might be left out, in particular due to the lack of case studies that demand supporting relationships among participants (FR10) or that employ home sensors, wearable devices, and JITAIs along with machine learning–based triggers [68,69]. These limitations are reflected in the 4-dimension planning framework, in the sensor dimension which might be more detailed to support such scenarios that demand, for instance, human activity recognition [70]. In future studies, new interviews can be conducted until new requirements are not identified [71]. In addition, we are running accessibility studies to provide solutions that can be used by different target-user profiles.

Concerning the 7-step-based process, the last step “results” should be extended to further assist data collection such as retrospective surveys [72], data analysis, and study reports. Besides, as data collection grows, integrating data analysis algorithms may support building predictive user models [69,73,74]. Further, with the growing adoption of experience sampling and program-based mHealth interventions, support to the specialists should be provided toward the production of reports in sufficient details to allow both replication and theory building, as was the case with randomized controlled trials [75].

Limitations of the ontology-based conceptual model include those identified in the postdesign interviews, which can be tackled by supporting participants’ records and by the organization of participants into groups and the organizing events into hierarchical groups. These and other features can be promoted with the formalization of the current integration with external services [57].

Furthermore, while our work included usability evaluations to improve both the method and the system, dealing with barriers highlighted by Byambasuren et al [47], and provided a complementary method for JITAIs guidelines, as proposed by Nahum-Shani et al [76], evaluations of mHealth interventions still are a prominent gap in the literature which ESPIM did not address, as precisely identified by Bradway et al [77] and Dick et al [78].

Finally, our study identified important nonfunctional requirements which have not been included in the method’s list of requirements, even though they are in consonance with recent literature and are partially attended by the current system. As a result of a systematic review on mHealth-related apps, Llorens-Vernet and Miró [79] offer a list of criteria, grouped into categories, aimed at guiding the development of mHealth apps. The systematic review ensured the categories (usability, privacy, security, appropriateness and suitability, transparency and content, safety, technical support and updates, and technology) are consistent with those identified by other authors in related contexts [7-10].

### Comparison With Prior Work

The ESM and EMA methods for mobile-based data collection are a popular alternative [25,80], while EMIs and JITAIs depend on mobile technologies to monitor contextual changings. Some contributions report authoring and deployment systems.

MyExperience [81] pioneered employing ESM via a customizable mobile app, supporting sensor data capture, contextual triggers configuration, and questionnaires authoring. The authors predicted several scenarios and inspired later works. However, ESM programs were added to mobile devices as XML files, and real-time monitoring was not possible. MyExperience’s spin-off movisensXS [32] is a platform with a graphical interface for creating questions with flow logic and media capture, temporal and contextual triggers, and real-time monitoring.

PACO (Personal Analytics COmpanion) [82] is an open-source system that allows the authoring of questions and media capture with branching features associated with logical operators. Despite its simplicity, PACO was the first to present an end-user graphical interface.

The ExperienceSampler [83] and AWARE [84] frameworks simplify the creation of ESM apps through logical and declarative programming. ExperienceSampler allows users to implement apps with messages, questions, skip and branching logic, and random-based notifications. AWARE focuses on logging sensor-based context information; its flexible contextual model considers 8 question types, branching logic, time, context-based notifications, and conditional broadcasts (eg, announce when each user answers a question).

Among commercially available systems, LifeData [31] enables the authoring of 14 types of tasks, including capturing photos and exhibiting a website, with logic and temporal flows, and self-initiated and random-based triggers. LifeData also enables the configuration of conditional actions and relationships among target users. Mobile EMA [33] provides a complex authoring interface, and allows displaying images in questions and integrating smartwatches to collect sensor data. LifeData and Mobile EMA systems comply with many of the functional requirements discussed in this paper, being extensively used in academic research [26-30].

Rough and Quigley [36] present recommendations and requirements for ESM authoring systems. They conducted interviews with 13 researchers and clinicians, 2 one-hour clinical observations, and 3 case studies with psychology researchers. They also propose the block-based visual programming tool Jeeves. The authors elicit 5 functional requirements for ESM tools: (1) collaboration and (2) support/share of projects, (3) tailoring of protocols and reminders to individuals, (4) debriefing/feedback/reminders in addition to surveys (ie, alternative types of tasks), and (5) ability to test both appearance and contextual behavior.

Investigating intervention design by specialists, Nahum-Shani et al [35] provided a framework for organizing theoretical and practical evidence into a model that supports authoring JITAIs. The authors discussed approaches for defining elements including states of vulnerability/opportunity and receptivity, outcomes, and adaptation strategy. Later, Nahum-Shani et al [76] proposed key components and design principles for designing mHealth JITAIs. The authors remark that authoring demands specialists to be concerned with components such as content, media, types of signal, temporal and contextual opportunities, receptivity marks, among others.

These works are complementary to ESPIM as they guide aspects of interventions’ planning. ESPIM provides a step-by-step model and ontology that allows organizing mHealth intervention components considering requirements from ESM, EMA, EMIs, JITAIs, and long-term studies with specialists from diverse areas. Furthermore, the ESPIM system demonstrates the feasibility of developing an authoring system based on the method, encompassing imperative features and providing flexibility for integrating ecological and just-in-time interventions.

### Conclusions

The ESPIM comprises a list of requirements for mHealth experience sampling and intervention-based methods and systems, a 4-dimension planning framework, a 7-step-based process, and an ontology-based conceptual model. The ESPIM system encompasses web and mobile apps. Besides overseeing the planning of an intervention program, ESPIMs components guide the design of an ESPIM-based software platform as in our study. Moreover, current limitations point to further research as well as practical actions.

Eight case studies show that the ESPIM method and system allowed specialists to be the users who planned, created, and deployed interventions. The case studies encompassed interventions by professionals from psychology, gerontology, computer science, speech therapy, and occupational therapy. Specialists’ target users were parents of children diagnosed with autism spectrum disorder, older persons, university students, children, and older person’s caregivers. The specialists reported being able to create and conduct their studies without modifying their original design. A qualitative evaluation of the ontology-based conceptual model showed its compliance to the elicited functional requirements.

## Appendix

Multimedia Appendix 1 Evaluations protocols and tasks.

Multimedia Appendix 2 Semi-structured interview questions.

Multimedia Appendix 3 ESPIM system details.

## Abbreviations

EMA: ecological momentary assessment
EMI: ecological momentary intervention
ESM: experience sampling method
ESPIM: Experience Sampling and Programmed Intervention Method
FR: functional requirements
HCI: human–computer interaction
JITAI: just-in-time adaptive intervention
mHealth: mobile health
PACO: Personal Analytics COmpanion
UEQ: User Experience Questionnaire
UI: user interface
UX: user experience
