# Supplementary material for: Experience Sampling and Programmed Intervention Method and System for Planning, Authoring, and Deploying Mobile Health Interventions: Design and Case Reports
Source: J Med Internet Res. 2021 Jul 12;23(7):e24278. doi: 10.2196/24278 (PMC8314159; doi:10.2196/24278)
Supplement: Multimedia Appendix 1 [file jmir_v23i7e24278_app1.doc]

# Multimedia Appendix 1: Evaluation methods’ details

Textbox SM1. Heuristic Evaluation.

| Heuristic Evaluation is a method which consists of an inspection evaluation conducted by a group of Human Computer Interaction (HCI) specialists based on a set of criteria called heuristics. Nielsen and Molich (1990) [1] suggest a set of 10 heuristics to guide the evaluation: Visibility of system status; Match between system and the real world; User control and freedom; Consistency and standards; Error prevention; Help users to recognize, diagnose, and recover from errors; Recognition rather than recall; Flexibility and efficiency of use; Aesthetic and minimalist design, and Help and documentation. For each identified violation, a severity rating is assigned: Not important (0): It is not a usability problem; Cosmetic (1): It should not be fixed unless extra time is available; Minor (2): Low priority problems that can be repaired; Major (3): High priority problem that should be repaired; Catastrophic (4): Serious problem that must be repaired. Each usability specialist first completes the analysis alone. Next, as a group, they consolidate their violation lists and produce a final report which details the problems with severity rates. |
| --- |

Textbox SM2. Usability Test.

| A Usability Test (Nielsen, 1994) [2] is an empirical method of evaluation which relies on direct observations of users' interaction with a system. In this test, user's performance and preferences are evaluated, which enables obtaining quantifiable measures of an established criterion by determining the maximum, minimum and desired limits of these measures. The test also allows evaluating factors that characterize the usability of a software, such as learnability, ease of use, efficiency, productivity, user satisfaction, flexibility, and safety in use. |
| --- |

Textbox SM3. Semantic Differential (SD) scale.

| The Semantic Differential scale (SD) [3] supports analyzing users' perceptions of different aspects of the system during interaction. Users judged a series of concepts against a series of bipolar, seven-step scales defined by bipolar adjectives by putting a checkmark in the appropriate position. Each one of the seven steps in the scale is assigned with a score ranging from -3 to +3. Negative numbers correlate to a general negative evaluation of the task, positive numbers to positive evaluations, and zero to a neutral evaluation. In this work, the bipolar adjectives correspond to a Portuguese translation of the User Experience Questionnaire – UEQ (Textbox SM4). |
| --- |

Textbox SM4. User Experience Questionnaire (UEQ).

| The User Experience Questionnaire (UEQ) is an end-user questionnaire designed to obtain a comprehensive impression of the user experience of a product in a simple and immediate way. Its design adopted an empirical approach for item selection towards ensuring practical relevance. Usability specialists collected terms and statements relative to both soft and hard aspects of usability and user experience. The design led to a 26-item questionnaire comprising, as factors, Attractiveness, Perspicuity, Efficiency, Dependability, Stimulation, and Novelty [4] (Figure SM3). |
| --- |

Textbox SM5. Protocol employed in evaluations.

| The protocol comprises:   1. Signature of the informed consent form and authorization for capturing image and sound, 2. Brief explanation about the usability test and about the ESPIM system, 3. Interaction with the system following a set of predefined tasks of a fictitious scenario, 4. Application of a survey containing questions about profile, experience with software for data collection and interventions, questions of the UEQ (Textbox SM4) and about usability aspects (SUS-based questionnaire) (Figures SM4 e SM5), 5. Semi-structured interview. |
| --- |

Table SM1. Scenario and tasks used in the first usability test (web application).

| **Scenario** | | |
| --- | --- | --- |
|  | A geriatrician plans a mHealth intervention for an older person (“participant”) who, having her cognitive performance affected, is assisted by a caregiver. For the time of the intervention, the geriatrician has the support of the caregiver (as an “observer”) to ensure the planned activities are carried out. The tasks performed in the system by the geriatrician are as follows. | |
| **Task** | | |
|  | **1** | Log in to the system |
|  | **2** | Add a “participant” |
|  | **3** | Create an intervention program a specific week |
|  | **4** | Add the caregiver as an “observer” |
|  | **5** | Add a specific person as a “participant” |
|  | **6** | Add an "Event" that triggers every day at 9:00 AM |
|  | **7** | Set up interventions as illustrated in the diagram (Figure SM1) |
|  | **8** | Save the created “event” and “program” |


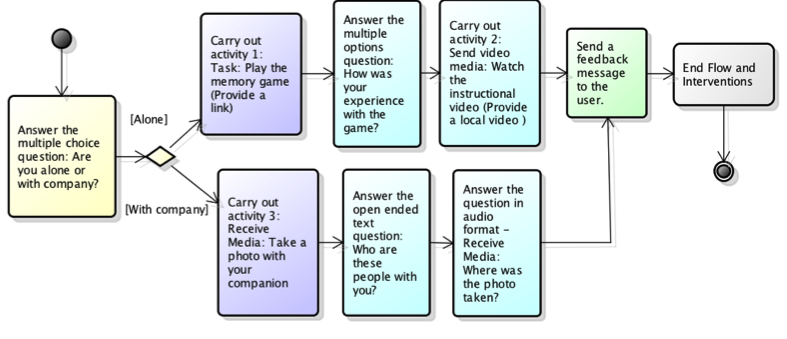


Figure SM1. Diagram with interventions proposed in Task 7 of the hypothetical scenario in geriatrics (Table SM1).


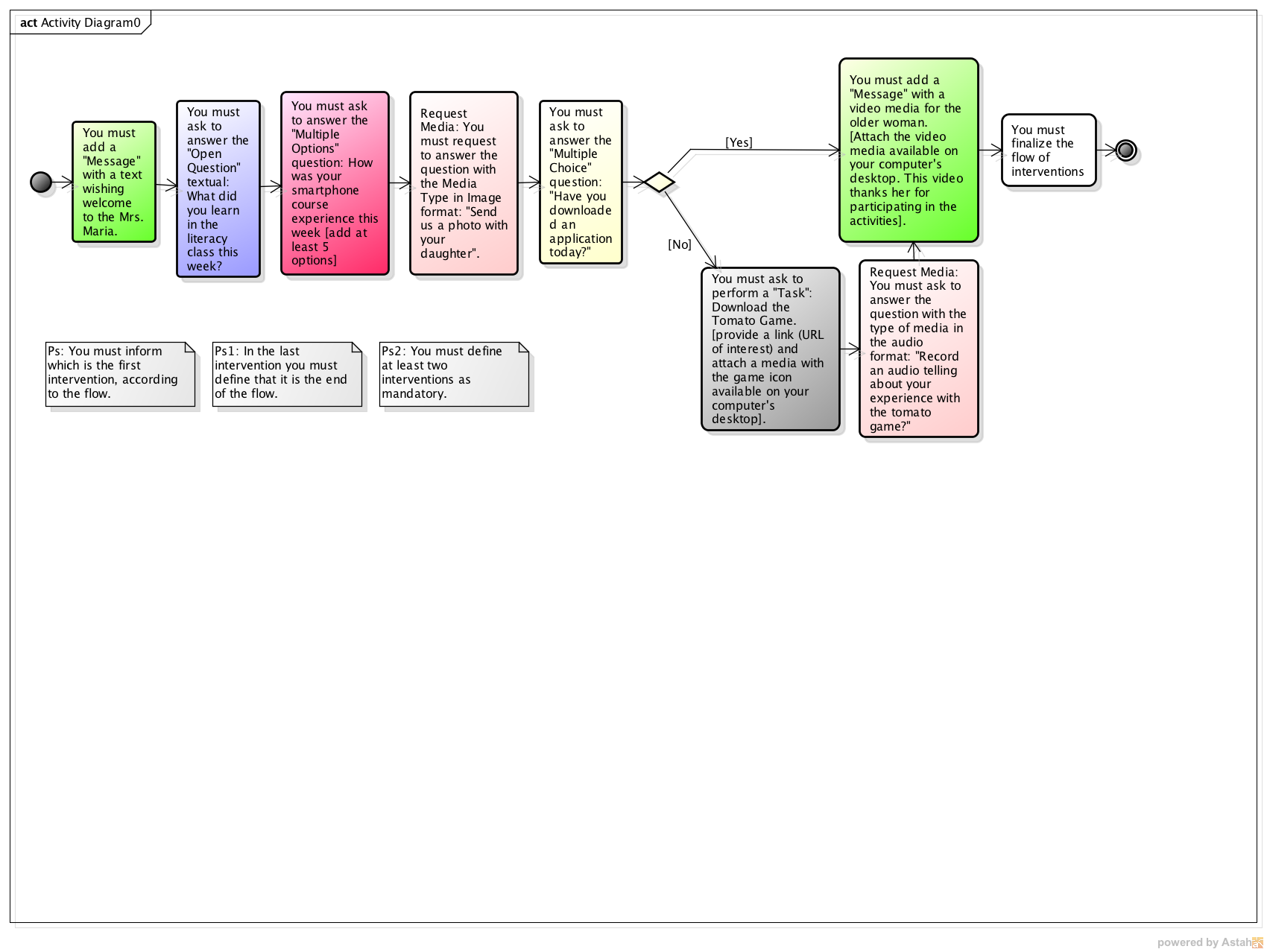


Figure SM2. Diagram with interventions proposed in Task 7 of the hypothetical scenario (Table SM2).

Table SM2. Scenario and tasks of the second usability test (web application).

| **Scenario** | | |
| --- | --- | --- |
|  | A gerontologist plans a mHealth intervention for an older person (“participant”) who participates in digital literacy classes. The older woman has some vision difficulties due to age and memory difficulties. During the course, the monitors will use an auxiliary application to help Mrs. Maria and the other older people to do daily activities and thus reinforce the learning of the content given in the classroom. For the time of the intervention, the gerontologist has the support of two monitors to ensure the planned activities are carried out and, also, of a Mrs. Maria's daughter (all as an “observers”). The tasks performed in the system by the gerontologist are as follows. | |
| **Task** | | |
|  | **1** | Log in to the system using Gmail |
|  | **2** | Add a "Program" that sends alerts for a week - from 16/02/2018 at 09:00 until 23/02/2018 at 23:30h. Give the name and description for the program |
|  | **3** | Add the monitors KRHR and LFS as a “Observers” |
|  | **4** | Add Mrs. Maria's daughter as an “observer”. You need create a email for her (xxxx@gmail.com) |
|  | **5** | Add Mrs. Maria as “Participant” |
|  | **6** | Add an "Event" with name and description |
|  | **7** | Set up interventions as illustrated in the diagram (Figure SM2 ) |
|  | **8** | Add "triggers" to Mrs. Maria's event for every day at 8 am. The type of sound must be a short notification and the time to display the alert about 30 minutes |
|  | **9** | Save the created “event” |
|  | **10** | Save the created “program” |

Figure SM3 - Portuguese version of UEQ [4].


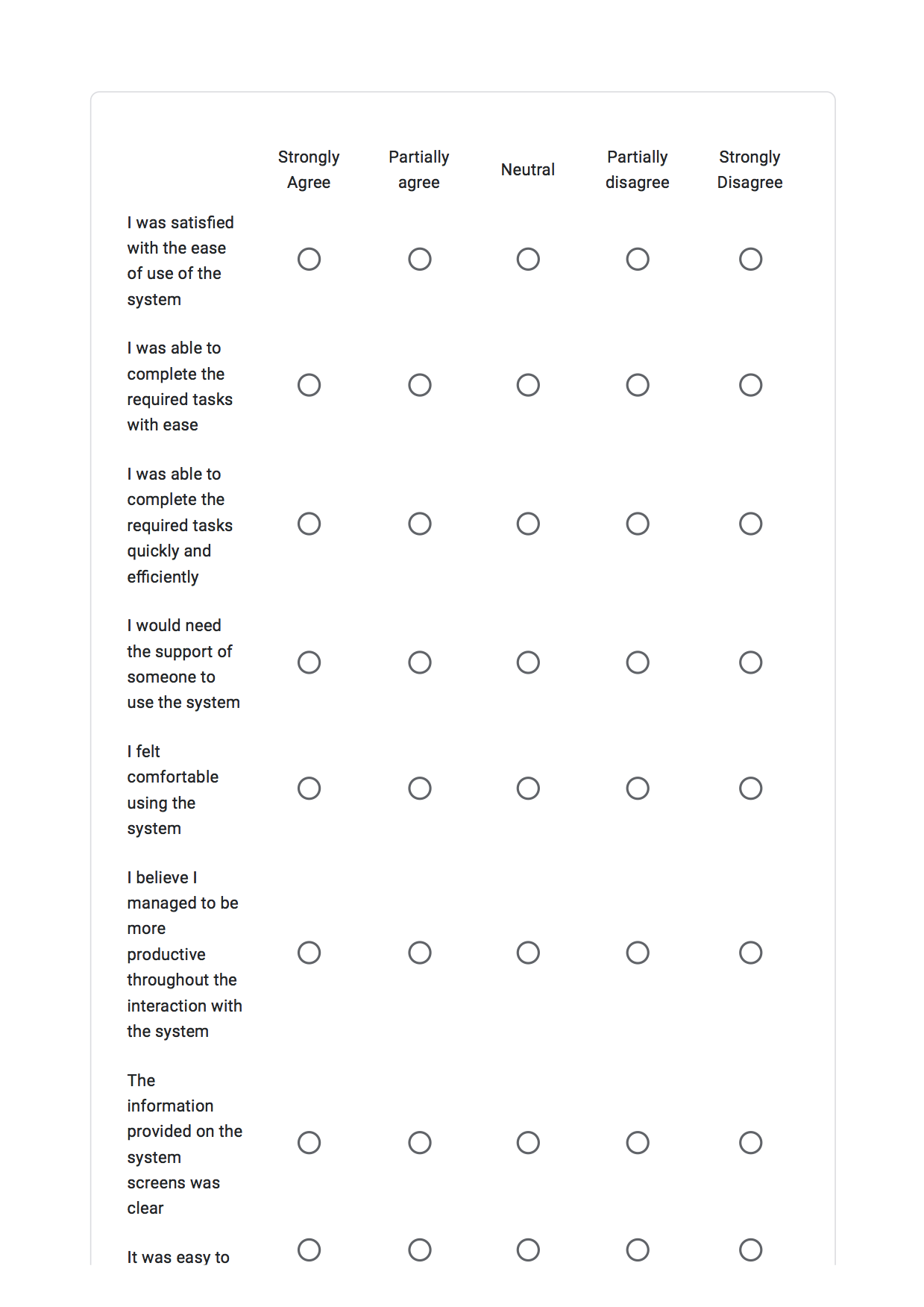


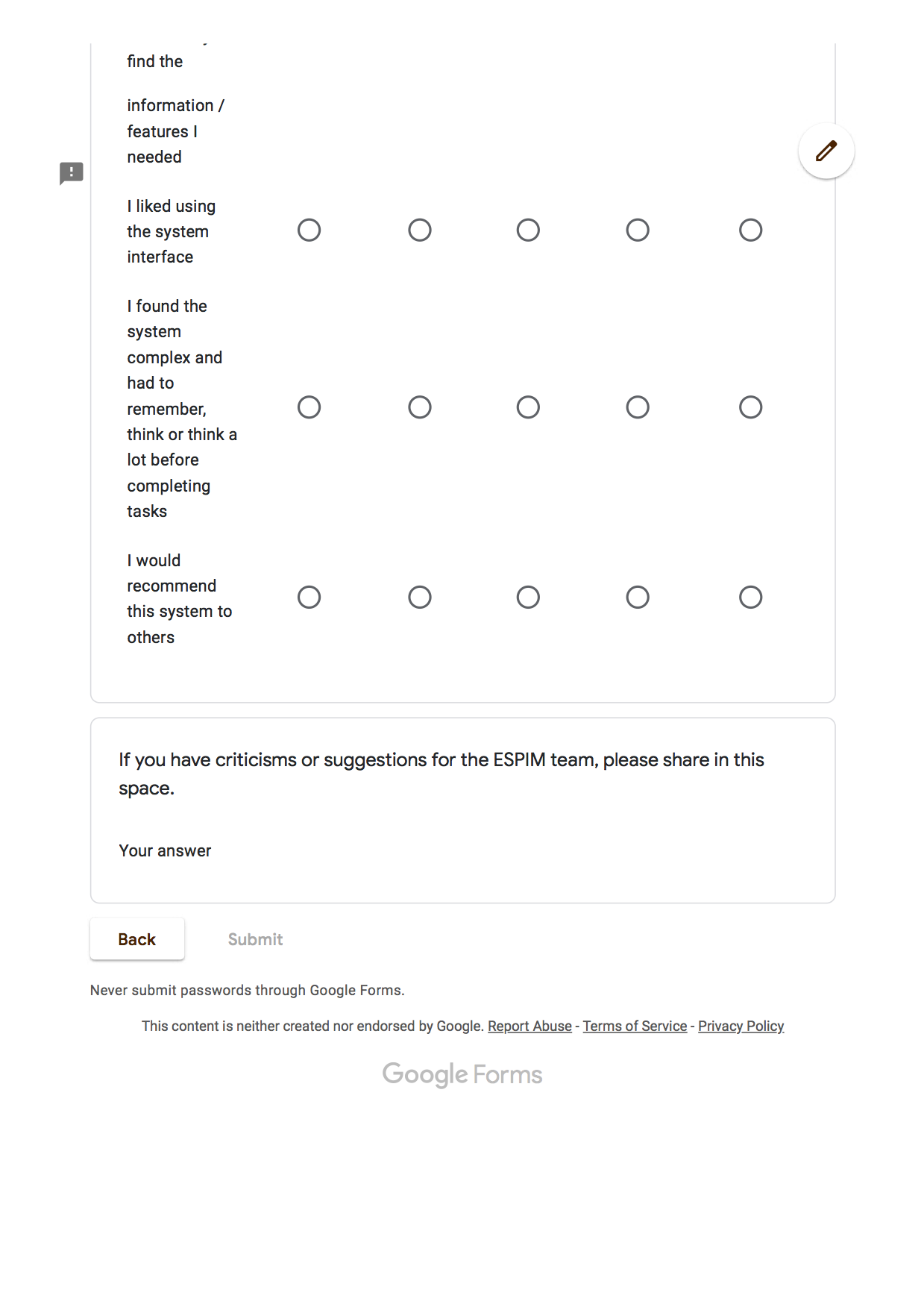


Figure SM4 – Questionnaire based on a Portuguese version of SUS [5].


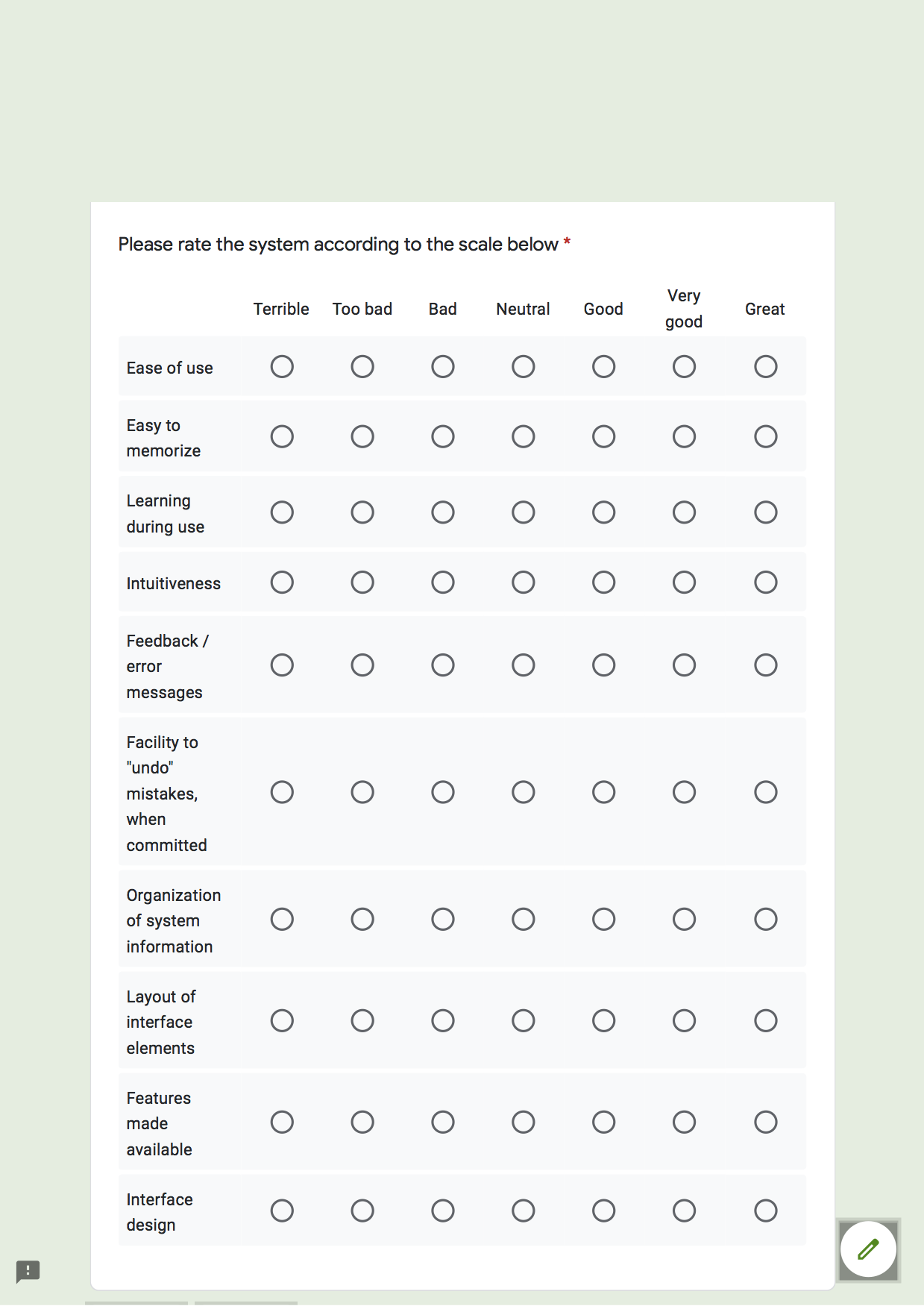


Figure SM5 – Questionnaire about usability based on Nielsen’s Heuristic [1].

## References

1.         Nielsen J, Molich R. Heuristic evaluation of user interfaces. Conference on Human Factors in Computing Systems - Proceedings [Internet] New York, New York, USA: Association for Computing Machinery; 1990 [cited 2020 Sep 9]. p. 249–256. [doi: 10.1145/97243.97281]

2.         Nielsen J. Usability inspection methods. Conference on Human Factors in Computing Systems - Proceedings [Internet] New York, New York, USA: Association for Computing Machinery; 1994 [cited 2020 Sep 9]. p. 413–414. [doi: 10.1145/259963.260531]

3.         Osgood CE, Suci GJ, Tannenbaum PH. The Measurement of Meaning. Urbana. University of Illinois Press; 1957.

4.         Laugwitz B, Held T, Schrepp M. Construction and Evaluation of a User Experience Questionnaire. Springer, Berlin, Heidelberg; 2008 [cited 2020 Sep 9]. p. 63–76. [doi: 10.1007/978-3-540-89350-9_6].

5. Brooke, J. SUS: A "quick and dirty" usability scale. In P. W. Jordan, B. Thomas, B. A. Weerdmeester, & A. L. McClelland (Eds.), Usability Evaluation in Industry, 1996. London: Taylor and Francis.
