# Supplementary material for: Experience Sampling and Programmed Intervention Method and System for Planning, Authoring, and Deploying Mobile Health Interventions: Design and Case Reports
Source: J Med Internet Res. 2021 Jul 12;23(7):e24278. doi: 10.2196/24278 (PMC8314159; doi:10.2196/24278)
Supplement: Multimedia Appendix 2 [file jmir_v23i7e24278_app2.doc]

# Multimedia Appendix 2: Semi-structured interview questions

O objetivo deste estudo é determinar se a modelagem computacional da metodologia ESPIM é adequada em diferentes aplicações nas áreas de atuação dos usuários especialistas.

Inicialmente vamos realizar uma Entrevista Estruturada para prover uma maior compreensão da sua área e das dificuldades encontradas na realização de sua pesquisa ou acompanhamento utilizando o ferramental ESPIM.

Dependendo dos resultados da entrevista, pode ser necessário um segundo encontro para alinhar as limitações e necessidades identificadas com as soluções determinadas pela pesquisadora responsável. Neste caso, os resultados desta primeira etapa serão utilizados para a criação de uma entrevista personalizada semiestruturada.

A sua participação no estudo é muito importante para determinar as limitações do modelo e de suas provas de contexto - a ferramenta de autoria e o aplicativo reprodutor de programas ESPIM - e se essas limitações estão associadas à modelagem ou a limitações das ferramentas.

Esta entrevista vários detalhes sobre sua pesquisa e utilização do sistema, por isso é importante que você esteja com um notebook com acesso ao seu material para consultar estes detalhes. Quanto maior o detalhamento melhor será a compreensão da situação ou problema envolvido. As questões desta entrevista serão disponibilizadas a todos os especialistas respondentes antes do encontro para que seja possível organizar o material e anotações necessárias para facilitar as respostas.

**Nome completo:**

**Link do currículo lattes:**

**PARTE 1**: Área de atuação e estudos relacionados. Esta seção é importante para indicar os conhecimentos e áreas de atuação do participante e o objetivo da utilização do sistema ESPIM nesse contexto.

1-) Resumo da formação acadêmica: graduação e pós-graduação (se possuir) com as respectivas instituições de ensino e áreas de pesquisa.

2-) Possui experiência profissional na área de formação? Em caso positivo resuma brevemente.

3-) Você utiliza ou utilizou o sistema ESPIM no contexto de pesquisa acadêmica? Em caso positivo: Qual o contexto (mestrado, doutorado ou pós-doutorado)? Resuma seu projeto de pesquisa.

4-) Você utiliza ou utilizou o sistema ESPIM no exercício de sua profissão? Em caso positivo: Qual a sua profissão? Resuma qual o seu trabalho no âmbito da utilização do sistema.

5-) Faça um resumo do(s) seu(s) estudo(s) realizado(s) utilizando o sistema ESPIM. Explique a motivação, objetivos, metodologia e quantidade e perfil de participantes (caso o estudo ainda não tenha sido executado você pode resumir o seu planejamento).

6-) Qual a relação do(s) estudo(s) conduzido(s) utilizando ESPIM com o seu/sua trabalho/pesquisa (acadêmico ou profissional)?

7-) Quais foram os seus resultados? Você tem alguma crítica com relação aos benefícios e/ou possíveis prejuízos da utilização da metodologia e do sistema ESPIM em seu estudo?

**PARTE 2**: Modelagem das intervenções. O objetivo desta parte é compreender como os seus programas interventivos foram criados e como isso foi realizado no sistema. O respondente pode anexar imagens associadas às intervenções para auxiliar os exemplos.

**Tempo de uso do sistema ESPIM:**

**Execução:**

1-) Para realizar o(s) estudo(s), como foi feita a criação do(s) programa(s) na interface Web do ESPIM? Descreva o(s) programa(s) em termos de conteúdo das intervenções e objetivos de coleta e/ou intervenção.

Descrição, objetivo e duração do **Programa**:

**Observadores** (outros além de você?):

**Participantes** (quantidade, perfil):

**Eventos** (quantidade, horários, frequência, tipo de alarme):

**Intervenções** (quantidade, tipos, conteúdos):

2-) Quais os tipos de intervenções disponíveis que foram utilizados (mensagem, questão aberta, questão de escolha única, questão de múltiplas escolhas, solicitação de mídia, tarefa)? Por que estas intervenções foram utilizadas? Dê um exemplo.

3-) Quais os recursos multimídia foram utilizados (áudio, vídeo e imagem) na captura (solicitação de mídia)? Por que estes recursos foram utilizados? Dê um exemplo.

4-) Quais recursos multimídia foram utilizados (áudio, vídeo e imagem) na visualização? Por que estes recursos foram utilizados? Dê um exemplo.

**Limitações:**

5-) Foi possível criar o(s) programa(s) ESPIM com o conteúdo que havia sido planejado para o seu(s) estudo(s)? Foram necessárias adaptações? Quais?

6-) Ao utilizar a interface de autoria Web, quais foram as dificuldades encontradas para transformar o seu planejamento em um programa ESPIM?

7-) Ao modelar os programas de acordo com os objetivos do seu experimento, quais foram as limitações encontradas? Por quê? Explique as limitações, exemplificando o que você gostaria de planejar porque não foi possível.

8-) As limitações e as adaptações realizadas tiveram consequências para o(s) estudo(s) e/ou para você? Por quê?

**Considerando o sistema ESPIM como um todo, ou seja, a interface Web e o aplicativo SENSEM:**

9-) Considerando as necessidades do meu estudo, o sistema ESPIM:

[ ] Já é suficiente para conduzir meus estudos da forma que planejei.

[ ] Precisa de algumas melhorias, mas consigo conduzir meus estudos na versão atual.

[ ] Precisa de algumas novas funcionalidades para que eu consiga conduzir meus estudos da forma que planejei, mas consigo conduzi-los com algumas adaptações.

[ ] Precisa de muitas novas funcionalidades, pois não consigo conduzir meus estudos em sua forma atual mesmo com adaptações.

Comentários:

10-) Com relação termos utilizados no ESPIM:

[ ] São de fácil compreensão: consegui entender e adaptar seu significado no contexto dos meus estudos nas primeiras utilizações do sistema.

[ ] São de média compreensão: consegui entender e adaptar seu significado no contexto dos meus estudos após utilizar o sistema por um tempo.

[ ] São complexos: tive dificuldade de entender e adaptar seu significado no contexto dos meus estudos, mas após utilizar o sistema por um tempo me acostumei.

[ ] São muito complexos: não consigo entendê-los e adaptá-los ao contexto dos meus estudos mesmo após utilizar o sistema por muito tempo.

Comentários:
