# Supplementary material for: Experience Sampling and Programmed Intervention Method and System for Planning, Authoring, and Deploying Mobile Health Interventions: Design and Case Reports
Source: J Med Internet Res. 2021 Jul 12;23(7):e24278. doi: 10.2196/24278 (PMC8314159; doi:10.2196/24278)
Supplement: Multimedia Appendix 3 [file jmir_v23i7e24278_app3.doc]

# Multimedia Appendix 3: ESPIM Software details

Besides guiding the design of an Intervention Program, ESPIMs apparatus can be used in the design of an ESPIM-based software platform such as the one built in our study. The iterative design leading to the ESPIM method involved the corresponding iterative prototyping of a software instance whose current version is illustrated in this section. For brevity, we present the main interfaces used by specialists when authoring (Figures SM1 to SM4), monitoring (Figure SM5) an Intervention Program, and an illustrative interface of the mobile app used by Participants (Figures SM6 and SM7).

Upon successful login in the ESPIM web, the specialist has options to list or edit Participants and Intervention Programs (Programs), and to access Results. Choosing Programs allows creating a new program (Figure SM1). Editing a new program allows providing Intervention Program Name, and optional Description and Starting and Ending dates (Figure SM1A to C). It is also possible to import an existing program, for reuse (Figure SM1D). The specialist navigates among the steps using proceed (Figure SM1E) and back options. In the ESPIM software, we adopted the following: identifying the program by a name is mandatory and accompanying textual description is optional; there is an option to export the program.

Also, in the current version it is not possible to organize a program in phases, requiring specialists to create an individual program for each stage. A future implementation may allow phases and automatically monitor participants' progress according to defined conditions (eg, temporal, answers, tasks, and activity performance).

In the next step, the specialist includes other Observers (Figure SM1F). In the current ESPIM software, the person creating the program is herself an Observer. Future implementations may support other roles, including an assistant role in charge of creating and editing Intervention Programs in the software platform of choice.

Next, the specialist associates the Participants to the program (Figure SM1G). The current ESPIM software manages participants’ identity via the email registered with the mobile app. Moreover, each participant may be associated with more than one pseudonym or alias. This facilitates one participant to engage in different Intervention Programs performing distinct roles since, at the present, distinct participant roles are managed via distinct Intervention Programs.

The present ESPIM system does not allow defining the relationship among participants. Thus, different participant roles must be managed via distinct Intervention Programs. Furthermore, it is not possible, at the present, to directly import participants’ information from third-party sources.


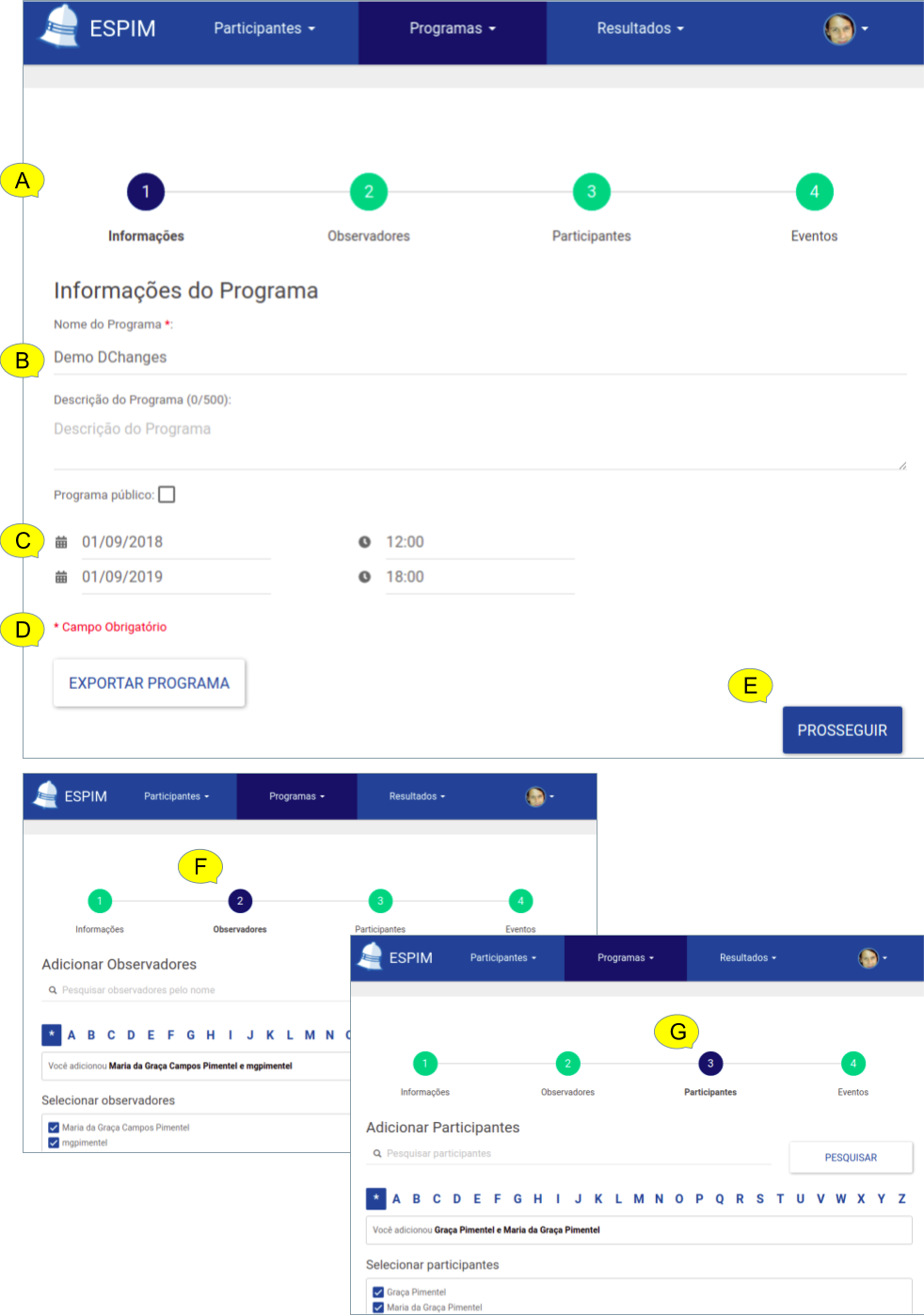


Figure SM1. ESPIM web application used by the specialist to create an Intervention Program. The first step (A) informs the program’s name and description (B), and duration (C). Options include exporting the program (D). The following steps register specialists (observers-F) and target-users (participants-G).

The step allowing the managing Events (Figure SM2A) allows specialists to edit events (B) by providing name, description, and color coding (Figure SM2C). It is possible to preview the text contained in each Intervention of its corresponding Intervention Flow (eg, a set of active tasks) (Figure SM2D) or to edit Intervention Flows (Figure SM2E).

The step also demands configuring triggers and alarms (Figure SM2F-G). Another option is configuring feedback to Observers toward reporting Participants (lack of) interaction with the Event. It is possible to create a new Event from scratch (Figure SM2I) or reusing an existing event with the import option (Figure SM2J). It is also possible to configure sensor-based sampling (Figure SM2K). Finalizing is always available (Figure SM2L). When the interaction corresponds to editing the Event’s Intervention Flow (Figure SM2E), the requested task is carried out in a new window (Figure SM3).

The present ESPIM software provides temporal and self-initiated triggers. Proximity-based contextual has been made available for one study. Random triggers and specialists-initiated have not been demanded by current studies and are not yet provided.


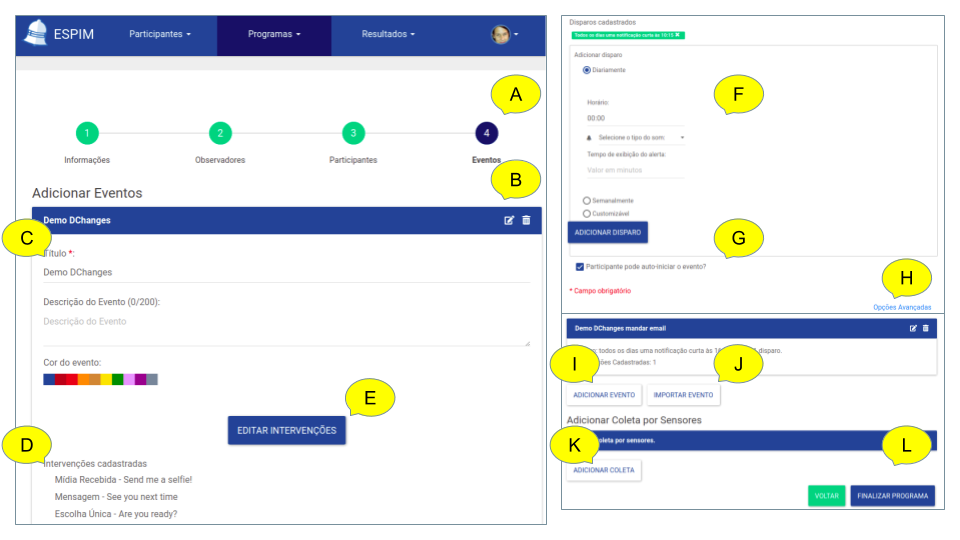


Figure SM2. ESPIM web application step for editing Events. In this step (A), specialists can edit events (B) providing name, description, and color coding (C). The text associated with existing interventions is also available (D). Specialists may edit Intervention Flows corresponding to the Event (E), configure time-based (F) and self-initiated (G) triggers and corresponding alarm types, or set up when observers are to be informed that participants interacted or missed an alarm (H). Options include creating a new Event from scratch (I) or via import (J). It is also possible to configure sensor-based sampling (K). Finalizing is always available (L).

The current ESPIM software has several types of Active Tasks including sending media-based instructions, requesting media-based responses, complex-condition and loop-based flows, and third-party app interaction activated with customized parameters.

Active tasks are associated with an Event (Figure SM2A) in a dedicated Intervention Flow interface (Figure SM3). Each task is specified as one intervention in the flow (Figure SM3 A-C). Each flow indicates the initial intervention (selected radio button in A). Each intervention may be indicated as mandatory or optional (checkbox in A-C). Moreover, each intervention may be presented to the participant using text (Figure SM3D) or other multimedia stimuli, or both. Text can be enhanced by markup. Multimedia stimuli include image (Figure SM3E), audio and video, which can be reused via upload (Figure SM4A) or recorded on the fly (Figure SM4B-C).

Among the several types of questions supported, the single-choice question (Figure SM3A) allows associating distinct interventions as the follow up of each alternative (Figure SM3F) as well as conditions to each question (eg, comparison with a predefined answer). An option allows associating any number of alternatives to multiple-choice and scale-based questions (Figure SM3G). A task-based intervention which requests the user to respond with some media (Figure SM3B) specifies the type of media collected among image, audio, or video (“image” in Figure SM3H). Each Intervention Flow must have at least on closing intervention (Figure SM3I), which is verified upon concluding the editing. The reuse of Intervention Flows is also supported via import and export options (Figure SM3J).


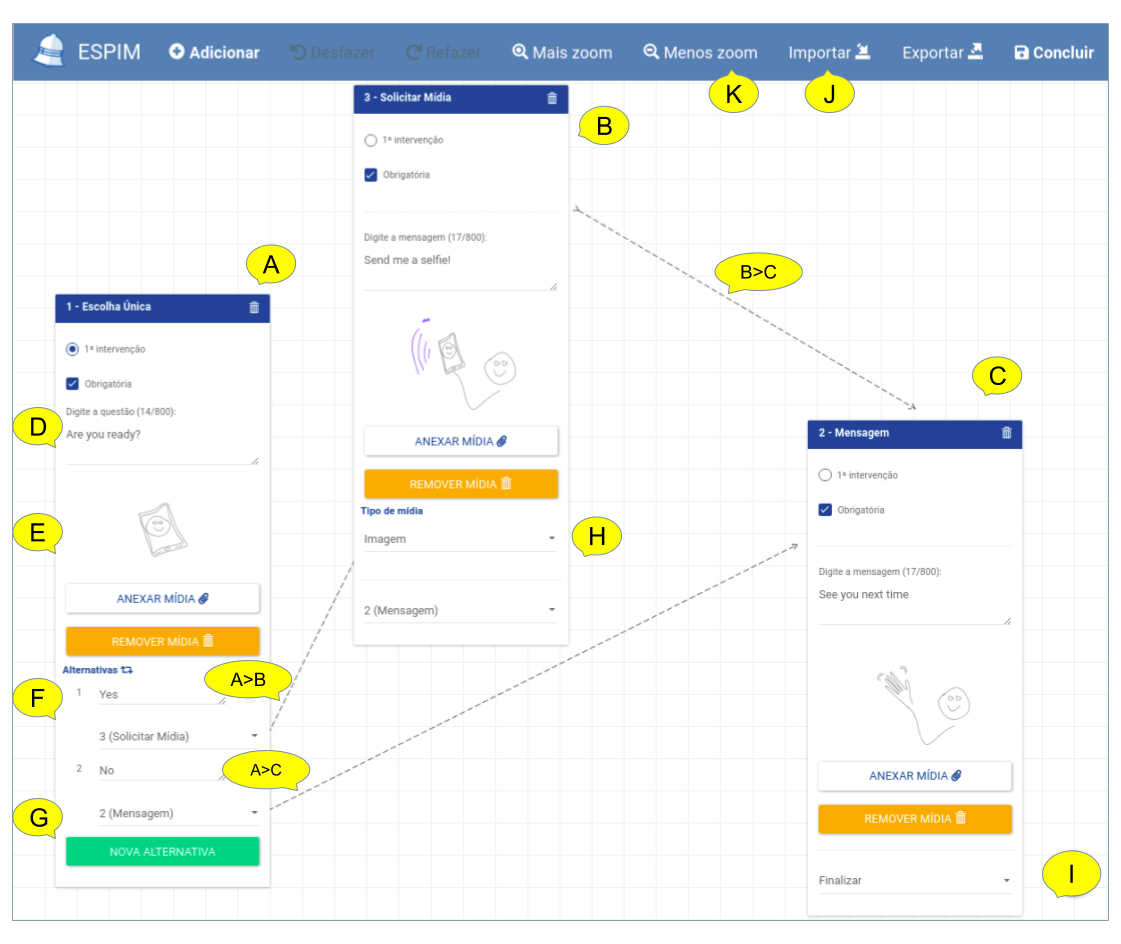


Figure SM3. ESPIM web interface to create an Active Task Flow. This flow contains three interventions: a single-choice question (A), a task-based intervention requesting an image (B), and a message intervention (C). Specialists may include instructions using text (D) or other media (image (E), audio or video), or both. They must indicate the initial intervention (radio button in A) and mark each intervention as mandatory or optional (checkbox in A-C). The app shows arrows to indicate the flow (eg B>C). In a single-choice intervention (F), specialists may associate specific interventions to each alternative (A>B and A>C). Also, they can add many alternatives of choice- and scale-based questions (G). When specialists create a task requesting media (B), they indicate the type of media required (“image” in H). They must nominate at least one closing intervention (I). The specialist can zoom (K), import and export (J) flows.


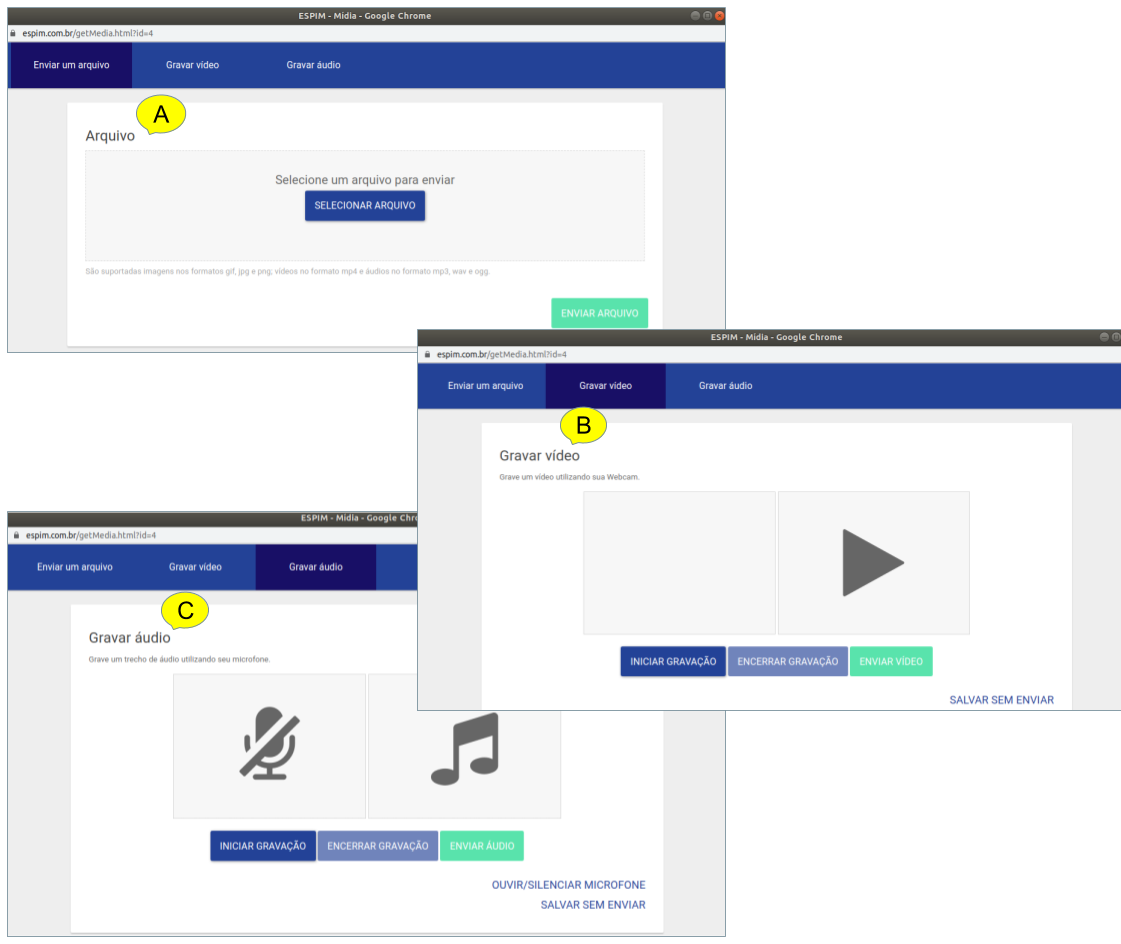


Figure SM4. ESPIM web interface to upload media-based stimuli (A) and to record video (B) or audio (C).


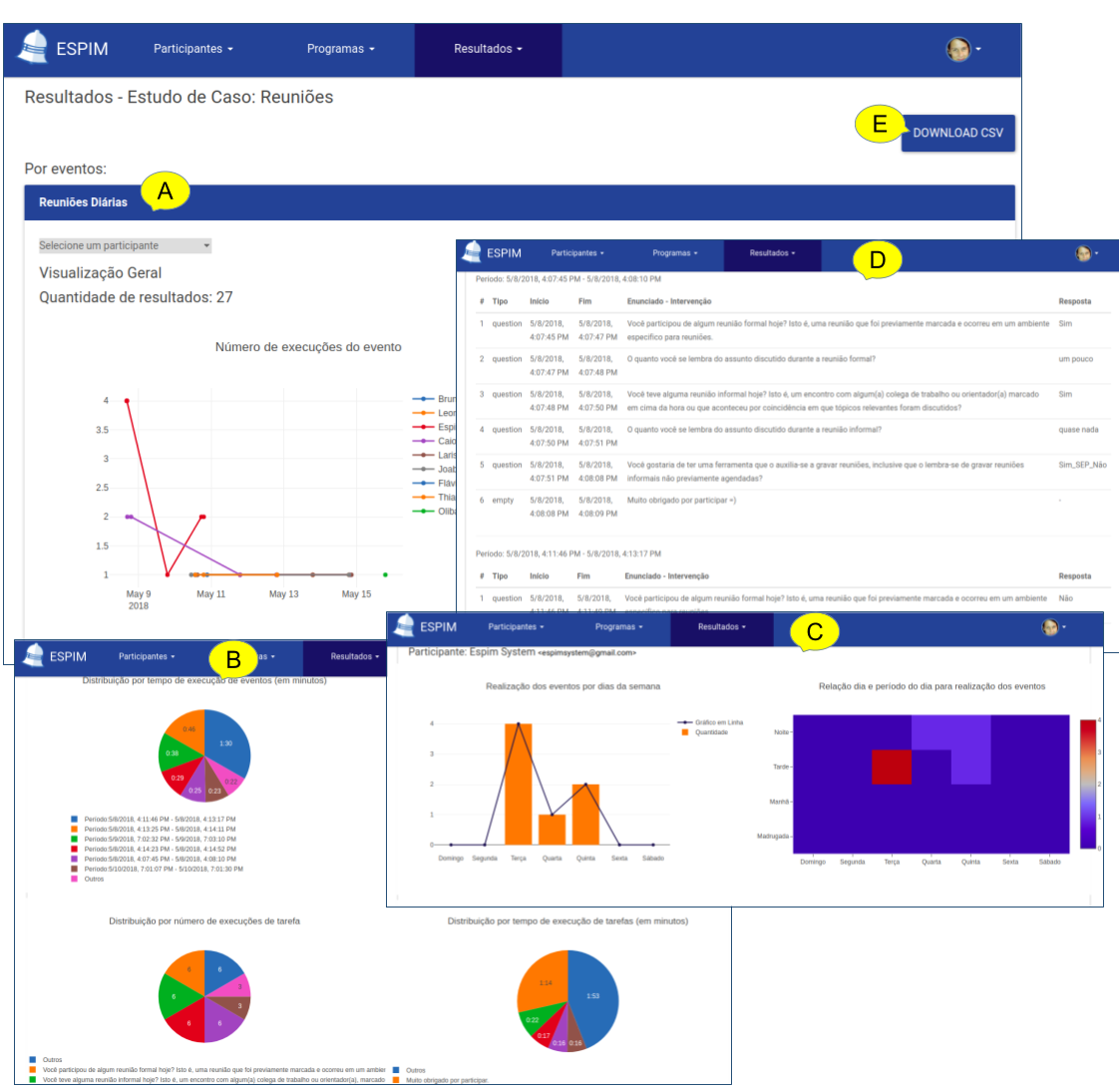


Figure SM5. ESPIM web interface to visualize results includes an overview by a participant (A), distributions of responses both per task (B) and along the time (C), access to individual responses (D), and an option for download (E).

Participants access the Event’s interventions by self-initiating the mobile app or by responding to a notification. The mobile app initiates to allow the participant to interact with the intervention flow (Figures SM6 and SM7).


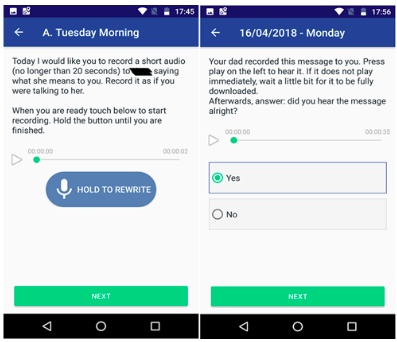


Figure SM6. ESPIM mobile app used by the target-users of an intervention requesting the user to record an audio message (left) and to respond to a single-choice question upon listening to an audio stimuli (right). Source: Zaine et al. (2019) [1].

| 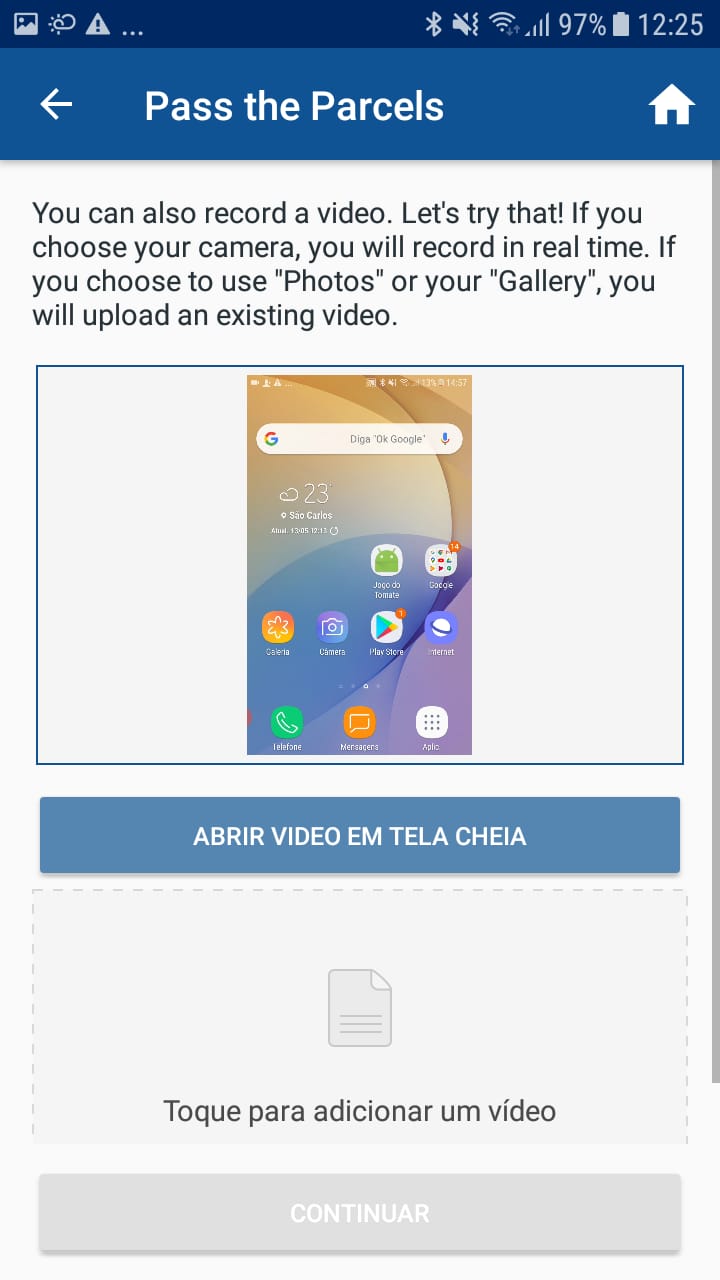 | 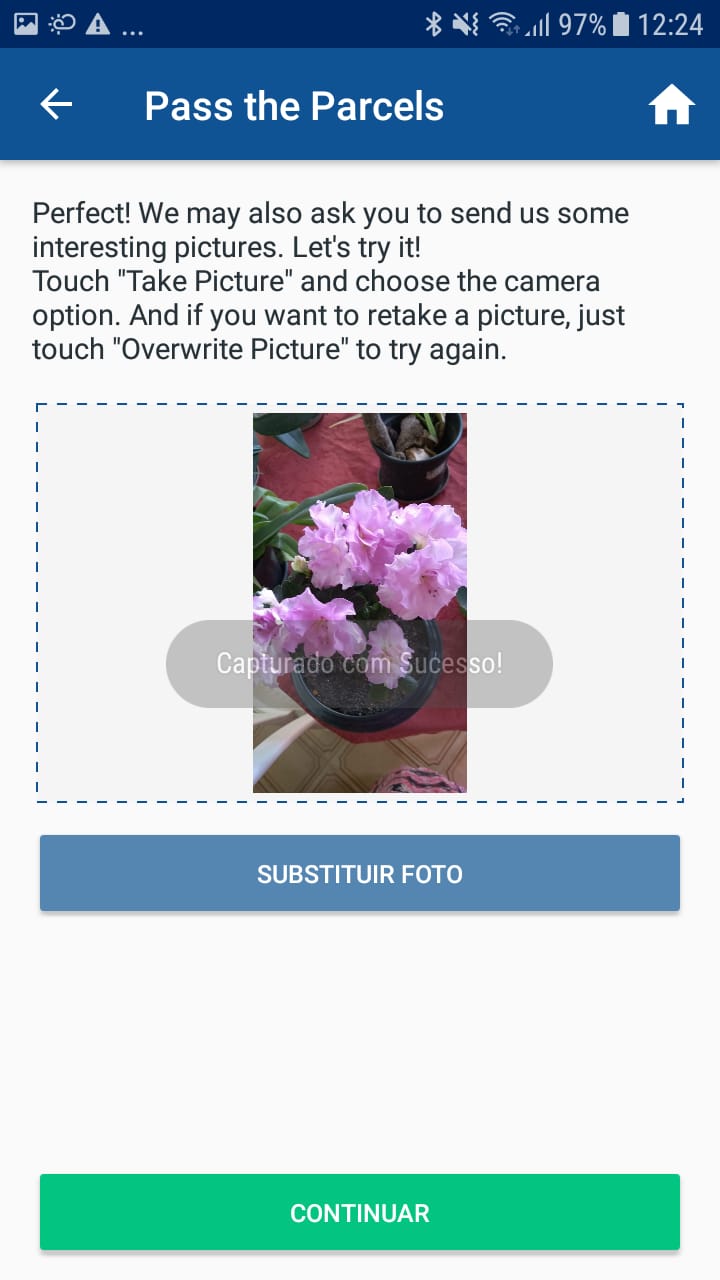 |
| --- | --- |

Figure SM7. ESPIM mobile app used by the target-users to watch and record a video (left) and to capture a picture (right).
